# Antimicrobial resistance point-of-care testing for gonorrhoea treatment regimens: cost-effectiveness and impact on ceftriaxone use of five hypothetical strategies compared with standard care in England sexual health clinics

**DOI:** 10.2807/1560-7917.ES.2020.25.43.1900402

**Published:** 2020-10-29

**Authors:** Emma M Harding-Esch, Susie E Huntington, Michael J Harvey, Georgie Weston, Claire E Broad, Elisabeth J Adams, S Tariq Sadiq

**Affiliations:** 1 Applied Diagnostic Research and Evaluation Unit, Institute for Infection and Immunity, St George’s University of London, London, United Kingdom; 2 National Infection Service, Public Health England, London, United Kingdom; 3 Aquarius Population Health, London, United Kingdom; 4 St George’s University Hospitals NHS Foundation Trust, London, United Kingdom

**Keywords:** Neisseria gonorrhoeae, Sexually transmitted infection, antimicrobial resistance, point-of-care test, cost-effectiveness, ceftriaxone, ciprofloxacin, azithromycin

## Abstract

**Background:**

Widespread ceftriaxone antimicrobial resistance (AMR) threatens *Neisseria gonorrhoeae* (NG) treatment, with few alternatives available. AMR point-of-care tests (AMR POCT) may enable alternative treatments, including abandoned regimens, sparing ceftriaxone use. We assessed cost-effectiveness of five hypothetical AMR POCT strategies: A-C included a second antibiotic alongside ceftriaxone; and D and E consisted of a single antibiotic alternative, compared with standard care (SC: ceftriaxone and azithromycin).

**Aim:**

Assess costs and effectiveness of AMR POCT strategies that optimise NG treatment and reduce ceftriaxone use.

**Methods:**

The five AMR POCT treatment strategies were compared using a decision tree model simulating 38,870 NG-diagnosed England sexual health clinic (SHC) attendees; A micro-costing approach, representing cost to the SHC (for 2015/16), was employed. Primary outcomes were: total costs; percentage of patients given optimal treatment (regimens curing NG, without AMR); percentage of patients given non-ceftriaxone optimal treatment; cost-effectiveness (cost per optimal treatment gained).

**Results:**

All strategies cost more than SC. Strategy B (azithromycin and ciprofloxacin (azithromycin preferred); dual therapy) avoided most suboptimal treatments (n = 48) but cost most to implement (GBP 4,093,844 (EUR 5,474,656)). Strategy D (azithromycin AMR POCT; monotherapy) was most cost-effective for both cost per optimal treatments gained (GBP 414.67 (EUR 554.53)) and per ceftriaxone-sparing treatment (GBP 11.29 (EUR 15.09)) but with treatment failures (n = 34) and suboptimal treatments (n = 706).

**Conclusions:**

AMR POCT may enable improved antibiotic stewardship, but require net health system investment. A small reduction in test cost would enable monotherapy AMR POCT strategies to be cost-saving.

## Introduction

Antimicrobial resistance (AMR) has developed to every class of antibiotic used for treatment of the bacterial sexually transmitted infection (STI) *Neisseria gonorrhoeae* (NG) [1], with increasing reports of multidrug-resistant strains [2]. NG, the second most prevalent bacterial STI globally [3], is associated with serious long-term reproductive health complications if left untreated.

World Health Organization (WHO) guidelines [4] recommend a treatment regimen that treats at least 95% of circulating NG strains, as monitored through antibiotic surveillance programmes such as Public Health England’s national Gonococcal Resistance to Antimicrobials Surveillance Programme (GRASP) [1]. Dual therapy with ceftriaxone and azithromycin is recommended in Europe [5], and was in the United Kingdom (UK) until 2019 [6] when it was replaced with 1 g ceftriaxone monotherapy due to the emergence of azithromycin resistance [7]. AMR to ceftriaxone, an extended-spectrum cephalosporin, is the most urgent threat [8,9] with few practical alternatives immediately available if widespread resistance develops.

Rapid diagnostics have been identified as a key approach to tackling AMR [10]. Rapid tests are those that have a two-hour turnaround, whereas point-of-care tests (POCTs) enable the test to be conducted, results obtained and treatment performed in the same clinical visit [11]. A principal feature of an NG AMR diagnostic is to assess antibiotic susceptibility at the time of NG diagnosis. A test that combines both NG diagnosis and AMR prediction at the point-of-care (AMR POCT) would allow the selection of appropriate treatment regimens for considerable numbers of NG infections, including safe use of antimicrobials which have been abandoned for widespread use due to circulating resistance, but which would be effective for a substantial proportion of infections [12]. For example, in the UK in 2018, 60% of NG infections were susceptible to ciprofloxacin, 90% to azithromycin and 88% to penicillin [1]. The ability to use these antibiotics to treat NG may in turn reduce AMR selection pressure on ceftriaxone [13].

Rapid tests are already being used for NG in some sexual health clinics (SHC) [14]. While laboratory-based NG fluoroquinolone susceptibility tests exist [15], rapid NG AMR tests are in development and being clinically evaluated. One such test is an NG fluoroquinolone susceptibility AMR POCT, developed within the Precise Study [16] using the io platform (Binx Health Limited (formerly Atlas Genetics), Boston, United States (US)) already CE-marked for *Chlamydia trachomatis* (CT) detection [12,17]. Costs and short-term clinical impacts of these tests are used in procuring sexual health services provision for a region (known as sexual health commissioning in England) and adoption into SHC decisionmaking [18].

In this analysis, we assessed the cost-effectiveness in English SHC of five hypothetical AMR POCT strategies for the treatment of NG, which enable use of ciprofloxacin and/or azithromycin, either alongside, or as an alternative to, ceftriaxone. Potential diagnostic resistance-determinants of these antibiotics are small in number (*gyrA* for ciprofloxacin; *23S rRNA* and *mtrCDE* transporter for azithromycin), are relatively well-understood, and their absence predictive of susceptibility (particularly for ciprofloxacin). The development of molecular AMR POCTs for detection of these determinants are thus technically feasible and therefore more likely to be immediately available [19-21].

## Methods

### Model structure

We compared standard care (SC) for NG treatment in the UK (at the time of investigation, ceftriaxone 500 mg and azithromycin 1 g dual therapy [6]) with five different AMR POCT strategies (Box, Supplementary Figure S1), where the AMR POCT was used as a reflex test to inform antibiotic selection irrespective of which test was used to diagnose NG initially. The AMR POCT strategies were chosen to either facilitate optimised choice of a second antibiotic alongside ceftriaxone (dual therapy), or enable a single antibiotic alternative to ceftriaxone (monotherapy) (Box and Table 1).

Box **Summary of antimicrobial resistance point-of-care test strategies**

**Standard care**

Standard care with dual therapy of intramuscular ceftriaxone (500 mg) and oral azithromycin (1 g single dose).

**Dual therapy, including ceftriaxone**

A) AMR POCT for ciprofloxacin resistance only. Infections identified as not resistant to ciprofloxacin are given oral ciprofloxacin (500 mg) plus ceftriaxone (500 mg). Infections identified as ciprofloxacin resistant are given SC.

B) Dual AMR POCT for azithromycin and ciprofloxacin resistance. If no azithromycin resistance is identified, SC is given. If azithromycin resistant, ciprofloxacin (500 mg) and ceftriaxone (500 mg) are given unless there is ciprofloxacin resistance, in which case ceftriaxone (500 mg) is given alone.

C) Dual AMR POCT for ciprofloxacin and azithromycin resistance. If no ciprofloxacin resistance is identified, ciprofloxacin (500 mg) and ceftriaxone (500 mg) are given. If ciprofloxacin resistant, SC is given, unless there is also azithromycin resistance, when ceftriaxone (500 mg) is given alone.

**Monotherapy optimisation**

D) AMR POCT for azithromycin resistance. If no azithromycin resistance is identified, azithromycin (2 g) is given. If azithromycin resistant, ceftriaxone (500 mg) and ciprofloxacin (500 mg) dual therapy is given. If the AMR POCT incorrectly shows no resistance (false negative for AMR), it is assumed the treatment fails. The treatment failure would be identified in the test-of-cure and the patient would then receive 500 mg ceftriaxone.

E) AMR POCT for ciprofloxacin. If no ciprofloxacin resistance is identified, 500 mg ciprofloxacin monotherapy is given. If ciprofloxacin resistant, SC is given. If the AMR POCT incorrectly shows no resistance, monotherapy is assumed to fail, the patient returns and receives 500 mg ceftriaxone alone.

AMR: amtimicrobial resistance; POCT: point-of-care test; SC: standard care.

**Table 1:** Summary of antimicrobial point-of-care test strategies

Strategy	Antibiotic(s) for which resistance is tested			Intended treatment regimen based on test result						
	A	+	B	No resistance to A			Resistance to A			Resistance to A + B
Strategy A	Ciprofloxacin	NA	NA	Ciprofloxacin	+	Ceftriaxone	**Azithromycin**	**+**	**Ceftriaxone**	NA
Strategy B	Azithromycin	+	Ciprofloxacin	**Azithromycin**	**+**	**Ceftriaxone**	Ciprofloxacin	+	Ceftriaxone	Ceftriaxone
Strategy C	Ciprofloxacin	+	Azithromycin	Ciprofloxacin	+	Ceftriaxone	**Azithromycin**	**+**	**Ceftriaxone**	Ceftriaxone
Strategy D	Azithromycin	NA	NA	Azithromycin^a,b^	NA	NA	Ciprofloxacin	+	Ceftriaxone	NA
Strategy E	Ciprofloxacin	NA	NA	Ciprofloxacin^b^	NA	NA	**Azithromycin**	**+**	**Ceftriaxone**	NA
Standard care	No resistance testing is done. Standard care is ceftriaxone 500 mg and azithromycin 1 g dual therapy [6].									

NA: not applicable.

^a^ Dose given: 2 g.

^b^ If incorrect test result and treatment fails, ceftriaxone is given.

Bold font indicates standard care antibiotics, i.e. azithromycin and ceftriaxone dual therapy.

Unless otherwise stated, doses are: ceftriaxone 500 mg; azithromycin 1 g; ciprofloxacin 500 mg.

The rationale for dual therapy strategies is based on the assumption that combination therapy is more effective at preventing emergence or spread of AMR and thereby preserves the use of ceftriaxone. The rationale for the monotherapy strategies is that an AMR POCT enables effective treatment of the known resistance profile, sparing the use of ceftriaxone [22].

Each strategy consisted of a series of intended treatment regimens, contingent on the results of the AMR POCT used. For example, in strategy B, the earliest intended treatment regimen was SC, where the AMR POCT indicated azithromycin resistance; the second intended treatment regimen was ceftriaxone and ciprofloxacin, where the AMR POCT then indicated ciprofloxacin resistance; the third intended treatment regimen was ceftriaxone monotherapy.

A decision tree model was constructed using TreeAge Pro version 2017 (TreeAge Software, Williamstown, United States (US)) to simulate a hypothetical cohort of 38,870 NG-diagnosed SHC attendees (21,915 men who have sex with men (MSM), 8,488 women and 8,467 men who have sex with women (MSW)), representing the total number of NG diagnoses in England SHC in 2015, obtained from the genitourinary medicine clinical activity dataset national surveillance data (GUMCAD) [23]. Our assumptions regarding AMR POCT use meant the model could not be used when considering presumptive treatment, e.g. for sexual contacts of NG-positive patients initially negative by microscopy but subsequently positive by nucleic acid amplification testing (NAAT). Approximately 10% of individuals diagnosed with NG are in contacts [24] but the epidemiological breakdown of these patients (e.g. women, MSW, MSM) and the nature of their NG diagnoses (e.g. microscopy negative and NAAT positive) is not reported. Therefore, contacts could not be removed from the hypothetical cohort.

Key model assumptions include: 100% compliance with test protocols; all patients entering the model are NG true-positives; dual AMR POCT results are available simultaneously; and there is no ceftriaxone resistance data (supported by England’s GRASP [1]) so patients with monotherapy treatment failure would return and be successfully treated with ceftriaxone only. Model assumptions are provided in Supplementary Table S1.

### Outcomes

We aimed to assess the costs and effectiveness of these AMR POCT strategies to optimise treatment regimen choice and reduce selection pressure on ceftriaxone. The primary outcomes were the total costs (2015/16 GBP(EUR)), the percentage of people given optimal treatment, and the percentage of people given non-ceftriaxone optimal treatment. Optimal treatment was defined as one which cured NG and did not contain an antibiotic against which there was resistance. Model definitions are provided in Supplementary Table S2. These data were used to calculate incremental cost-effectiveness ratios (ICER, see equation) for the cost per additional optimal treatment gained and the cost per additional ceftriaxone treatment avoided. This was chosen as the measure of cost-effectiveness rather than other measures, such as cost per Quality Adjusted Life Years (QALY), because little data exist on the consequence of optimal vs suboptimal NG treatment on long-term outcomes, such as mortality or lifetime costs.

$$ICER\;=\;\frac{Cost_{B}\;-\;Cost_{A}}{Effectiveness_{B}\;-\;Effectiveness_{A}}$$

A: standard care; B: antimicrobial resistance point-of-care test strategy; ICER: incremental cost-effectiveness ratio.

ICERs were calculated for two types of effectiveness: optimal treatments and ceftriaxone treatments avoided.

Secondary outcomes were the percentage of people given a missed earlier intended treatment regimen (MEITR), and the percentage of people failing treatment due to resistance. MEITR was defined as the use of a treatment regimen which cured NG, but where an earlier intended treatment regimen would have provided optimal treatment because susceptible infections had been misclassified as resistant by the AMR POCT. MEITRs were independent of treatment effectiveness.

### Treatment

AMR POCT strategy treatment regimens were developed with input from three senior clinicians at St George’s University Hospitals NHS Foundation Trust, London, who outlined current and hypothetical AMR POCT patient pathways (Supplementary Figure S1). The purpose of the work was to determine AMR POCT strategy for short-term clinical impacts, because these are the data used for sexual health service provisioning and decisionmaking for adoption into SHC [18]. Furthermore, progression to longer-term clinical impacts from suboptimally treated infection is poorly defined [25]. Therefore, the time horizon was that of initial patient treatment. Complications associated with STIs, such as pelvic inflammatory disease (PID) in women, and adverse drug events associated with treatment were not considered.

### Model parameters

Model epidemiology parameters are presented in Table 2, and cost parameters in Table 3 and Supplementary Table S3. The hypothetical AMR POCT sensitivity and specificity were based on other NAAT-based rapid and POC tests [26-28], and altered in sensitivity analyses. Antibiotic resistance prevalences were obtained from national surveillance of SHC attendees (GRASP, 2017) [29]. GRASP is England’s national sentinel surveillance system that detects and monitors AMR in NG and records potential treatment failures. As the time horizon was that of initial patient treatment, discounting rates were not applied.

**Table 2:** Epidemiology parameters used in the model for antimicrobial point-of-care test strategies

Variable		Percentage (%)						Number						Comments and references
		MSM		W		MSW		MSM		W		MSW		
		Base case value	Range (low, high)	Base case value	Range (low, high)	Base case value	Range (low, high)	Base case value	Range (low, high)	Base case value	Range (low, high)	Base case value	Range (low, high)	
1	Initial clinic attendances	56.4	NA	21.8	NA	21.8	NA	21,915	NA	8,488	NA	8,467	NA	GUMCAD, 2015 [23]
2	Resistance to azithromycin^a^	4.7	3.3–6.1	2.7	1.9–3.5	5.3	3.7–6.9	1,030	723–1,337	229	161–297	449	313–584	GRASP, 2017 [29]
3	Resistance to ceftriaxone	0	0–0	0	0–0	0	0–0	0	0–0	0	0–0	0	0–0	GRASP, 2017 [29]
4	Resistance to ciprofloxacin^b^	36.2	25.3–47.1	20.1	14.1–26.1	32.5	22.8–42.3	7,933	5,544–10,322	1,706	1,197–2,215	2,752	1,930–3,582	GRASP, 2017 [29]
5	Sensitivity of AMR POCT	98	90–100	98	90–100	98	90–100	NA	NA	NA	NA	NA	NA	Assumption
6	Specificity of AMR POCT	99	90–100	99	90–100	99	90–100	NA	NA	NA	NA	NA	NA	Assumption

AMR: antimicrobial resistance; GUMCAD: genitourinary medicine clinical activity dataset; GRASP: gonococcal resistance to antimicrobial surveillance programme; MSM: men who have sex with men; MSW: men who have sex with women; NA: not applicable; POCT: point-of-care test; W: women.

^a^ The azithromycin resistance ranges were extended further to 1–10% for all population groups in one-way azithromycin resistance analysis so that the effect of more extreme values could be explored.

^b^ The ciprofloxacin resistance ranges were extended further to 0–50% in one-way ciprofloxacin resistance analysis so that the effect of more extreme values could be explored.

**Table 3:** Cost parameters used in the model for antimicrobial point-of-care test strategies

Cost input	Cost^a^		Comments and references
	Base case value	Range (low–high)	
Management of NG (oral medication/IM injection)	GBP 53.00/ GBP 62.74 (EUR 70.88 / EUR 83.90)	GBP 37.10–68.90 / GBP 43.92–81.56 (EUR 49.6–92.1 / EUR 58.73–109.07)	^b^Adapted from previous model. Adams, 2014 [30]
Return visit due to treatment failure	GBP 48.01 (EUR 64.20)	GBP 33.61–62.41 (EUR 44.95–83.46)	^b,c^ Adapted from previous model. Adams, 2014 [30]
Single AMR POCT	GBP 29.00 (EUR 38.78)	GBP 20.00–40.00 (EUR 26.75–53.49)	Estimate [47]
Dual AMR POCT	GBP 31.90 (EUR 42.66)	GBP 29.00–58.00 (EUR 38.78–77.56)	Estimate – 10% more than price of single AMR POCT (multiplier 1.1, range 1.0–2.0)
Dual AMR POCT	GBP 31.90 (EUR 42.66)	GBP 22.00–44.00 (EUR 29.42–58.84)	Estimate – single AMR POCT is varied, multiplier remains at 1.1 (10% more than price of single AMR POCT)
Azithromycin 1 g^d^	GBP 1.16 (EUR 1.55)	GBP 0.81–1.51 (EUR 1.08–2.02)	BNF, 2016 [34]
Azithromycin 2 g^d^	GBP 2.32 (EUR 3.10)	GBP 1.62–3.02 (EUR 2.17–4.04)	BNF, 2016 [34]
Ceftriaxone 500 mg ^e^	GBP 9.58 (EUR 12.81)	GBP 6.71–12.45 (EUR 8.97–16.65)	BNF, 2016 [34]
Ciprofloxacin 500 mg^d^	GBP 0.07 (EUR 0.09)	GBP 0.05–0.09 (EUR 0.07–0.12)	BNF, 2016 [34]

AMR: antimicrobial resistance; BNF: British National Formulary; IM: intramuscular; NG: *Neisseria gonorrhoeae*; POCT: point-of-care test.

^a^ GBP costs were converted to EUR using a historic currency conversion of an average of 366 days from 1 July 2015 to 30 June 2016 [48]. For this time period, GBP 1 = EUR 1.34, and EUR 1 = GBP 0.75.

^b^ Includes staff time and consumables but not antibiotic costs. Costs were inflated to 2015/16 costs using the Hospital and Community Health Services (HCHS) Inflation Indices 2015 produced by the Personal Social Services Research Unit [33]. No data were available for inflation from 2014/15 to 2015/16 so it was assumed to be the same as between 2013/2014 and 2014/15. The United Kingdom hospital consumer price index for health services shows similar annual growth in this sector from 2014 (93.2 in 2013, 97.1 in 2014 and 100 in 2015), which validates this assumption [49]. GBP costs were converted to EUR using a historic currency conversion of an average of 366 days from 1 July 2015 to 30 June 2016 [48]. For this time period, GBP 1 = EUR 1.34, and EUR 1 = GBP 0.75. A further breakdown of cost data are provided in Supplementary Table S3.

^c^ Within the context of this model, treatment failure due to resistance to a monotherapy would result in a return visit. No repeat culture would be taken and no repeat diagnostic tests would occur. The patient would be successfully treated using ceftriaxone, administered via injection.

^d^ Oral medication.

^e^ Administered via intramuscular injection. The price quoted is for 1 g vial of ceftriaxone, the smallest non-proprietary vial available [34] - the remaining 500 mg is then discarded.

A micro-costing approach was employed, considering only costs incurred to the healthcare provider (i.e. SHC). Costs to those procuring sexual health services provision, or to health systems as a whole, were not considered. Costs were estimated by adapting an existing model [30] and included: laboratory equipment; POCTs and antibiotics; AMR POCTs; and NG treatment implementation (e.g. staff time and consumables, including partner notification and health promotion) (Supplementary Table S3). It was assumed the AMR POCTs produced results in 30 min (maximum acceptable POCT run-time for service users [31,32]) and that in all strategies, NG-positive samples would still be sent to the laboratory for culture and phenotypic resistance testing. Costs are given in 2015/16 prices (GBP (EUR)) and inflated when based on old estimates [33]. Antibiotic prices were extracted from the British National Formulary (BNF) website (September 2016), with the cheapest formulation being used including non-proprietary costs where available [34]. Initial costs of diagnosing NG were not considered as people only entered the model after an NG diagnosis. The cost of implementing a change to clinical practice was also not considered.

### Sensitivity analyses

We conducted one-way analyses using TreeAge Pro version 2017 (TreeAge Software) and R software version 3.5.0 (R Foundation, Vienna, Austria), for each of the model parameters by varying them independently at the ends of their ranges to examine the effect on the primary outcome (Table 2). These analyses identified which model parameters results were most sensitive to. Each sensitivity analysis compared one of the five AMR POCT strategies with SC, across three population groups (women, MSW, and MSM). Probabilistic sensitivity analyses (PSA) were not performed because our analysis was a cost-effectiveness analysis with the outcome as cost per event avoided, rather than a cost acceptability or cost utility analysis exploring the likelihood that the technology is cost-effective at different willingness to pay (WTP) thresholds. There is no commonly agreed WTP for our outcome, and therefore presenting PSA results would likely not have yielded additional beneficial information.

This report was written following the Consolidated Health Economic Evaluation Reporting Standards (CHEERS) checklist [35].

### Ethical statement

As the only data included for this study were nationally published surveillance data from PHE, and no patient data were used, ethical approval was not required.

## Results

Overall AMR POCT strategy costs, treatments used, and treatment outcomes compared with SC in all groups are presented in Table 4. Breakdowns by population group are presented in Supplementary Tables S4, S5 and S6.

**Table 4:** Total costs, treatments used and treatment outcomes for standard care and antimicrobial resistance point-of-care test strategies: all groups (n = 38,870)

Strategy	Total cost^a^	Number of antibiotics used to treat NG			Number of optimal treatments^b^	Number of suboptimal treatments^c^	Number of MEITR^d^	Number of treatment failures^e^
		Ceftriaxone	Azithromycin	Ciprofloxacin				
Standard care	GBP 2,856,168 (EUR 3,819,524)	38,870	38,870	0	37,162	1,708	NA	NA
A: Single POCT for ciprofloxacin; dual therapy	GBP 3,954,554 (EUR 5,288,385)	38,870	12,408	26,462	38,057	813	265	NA
B: Dual POCT for azithromycin and ciprofloxacin; dual therapy	GBP 4,093,844 (EUR 5,474,656)	38,870	36,825	1,373	38,822	48	267	NA
C: Dual POCT for ciprofloxacin and azithromycin; dual therapy	GBP 4,066,498 (EUR 5,438,086)	38,870	11,736	26,462	38,611	259	912	NA
D: Single POCT for azithromycin; monotherapy	GBP 3,271,684 (EUR 4,375,189)	2,080	36,825	2,045	38,164	706	372	34
E: Single POCT for ciprofloxacin; monotherapy	GBP 3,457,581 (EUR 4,623,788)	12,656	12,408	26,462	38,057	813	265	248

AMR: antimicrobial resistance; MEITR: missed earlier intended treatment regimen; NG: *Neisseria gonorrhoeae*; POCT: point-of-care test.

^a^ GBP costs were converted to EUR using a historic currency conversion of an average of 366 days from 1 July 2015 to 30 June 2016 [48]. For this time period, GBP 1 = EUR 1.34 , and EUR 1 =  GBP 0.75.

^b^ ‘Optimal’ refers to a treatment regimen which cures the NG infection and does not contain any antibiotic against which there is resistance.

^c^ ‘Suboptimal’ refers to a treatment regimen which contains antibiotics against which there is NG resistance - if the treatment is a monotherapy it will result in treatment failure.

^d^ ‘Missed earlier intended treatment regimen’ (MEITR) refers to a treatment regimen which cures the NG infection and does not contain any antibiotic against which there is resistance, but a treatment regimen was used when an earlier intended treatment regimen would have provided optimal treatment – a MEITR is due to a false-resistant AMR POCT result.

^e^ ‘Treatment failure’ refers to failure to cure an NG infection due to resistance to an antibiotic given as monotherapy and is due to a false-susceptible AMR POCT result.

### Costs

The cost of SC NG management was GBP 2,856,168 (EUR 3,819,524) for the total cohort (Table 4). All AMR POCT strategies cost more than SC, with dual therapy AMR POCT strategies more expensive than monotherapy strategies. Strategy D was the least expensive AMR POCT strategy, costing GBP 3,271,684 (EUR 4,375,189), 14.5% more than SC. Strategy B was the most expensive, costing GBP 4,093,844 (EUR 5,474,656), 43% more than SC. This was consistent across all population groups (Supplementary Tables S4, S5 and S6).

### Optimal treatment

All AMR POCT strategies provided more optimal treatments than SC, in all population groups. Strategy B provided most optimal (n = 38,822) and least suboptimal (n = 48) treatments. Strategies A and E equally provided the least optimal treatments and the most suboptimal (n = 813) (see Table 4 and Supplementary Tables S4, S5 and S6).

### Ceftriaxone-sparing treatments given

Since all dual therapy strategies used ceftriaxone, only monotherapy strategies provided ceftriaxone-sparing options. Strategy D reduced ceftriaxone use by 95% compared with SC (Table 4).

### MEITRs given

A MEITR refers to a treatment regimen being used when an earlier intended treatment regimen would have provided optimal treatment. In all population groups, the fewest were in Strategies A and E (n = 265), and B (n = 267), and the most were in Strategy C (n = 912) (Table 4, Supplementary Tables S4, S5 and S6).

### Treatment failures

There were some treatment failures in each monotherapy strategy due to false-susceptible AMR POCT results: strategy D had 34/38,870 (0.09% of treatments) and Strategy E had 248/38,870 (0.64% of treatments) (Table 4). There were no treatment failures with SC or dual therapy strategies (A, B and C) because they all included ceftriaxone. This was consistent across all population groups (Supplementary Tables S4, S5 and S6).

### Cost-effectiveness analysis

The cost-effectiveness analysis (CEA) results are presented in Table 5. When avoidance of suboptimal treatments was considered, Strategy D was most cost-effective relative to SC, costing GBP 414.67 (EUR 554.53) per optimal treatment gained. Strategy A was least cost-effective overall, whereas Strategy B was the most-cost effective dual therapy strategy.

**Table 5:** Cost effectiveness analysis for standard care and antimicrobial resistance point-of-care test strategies

Subgroup	Comparison	Total additional cost^a^	Additional cost per patient^a^	Number of optimal treatments gained	Additional cost per optimal treatment gained^a^	Number of ceftriaxone treatments avoided	Additional cost per ceftriaxone-sparing treatment^a^
All	AMR POCT A vs SC	GBP 1,098,386.00 (EUR 1,468,860.00)	GBP 28.26 (EUR 37.79)	895	GBP 1,226.97 (EUR 1,640.81)	0	Dominated
	AMR POCT B vs SC	GBP 1,237,676.00 (EUR 1,655,131.00)	GBP 31.84 (EUR 42.58)	1,660	GBP 745.44 (EUR 996.87)	0	Dominated
	AMR POCT C vs SC	GBP 1,210,330.00 (EUR 1,618,562.00)	GBP 31.14 (EUR 41.64)	1,449	GBP 835.39 (EUR 1,117.16)	0	Dominated
	AMR POCT D vs SC	GBP 415,516.00 (EUR 555,665.30)	GBP 10.69 (EUR 14.30)	1,002	GBP 414.67 (EUR 554.53)	36,790	GBP 11.29 (EUR 15.09)
	AMR POCT E vs SC	GBP 601,414.00 (EUR 804,264.80)	GBP 15.47 (EUR 20.69)	895	GBP 671.82 (EUR 898.42)	26,214	GBP 22.94 (EUR 30.68)
MSM	AMR POCT A vs SC	GBP 620,274.00 (EUR 829,486.10)	GBP 28.30 (EUR 37.85)	499	GBP 1,242.13 (EUR 1,661.09)	0	Dominated
	AMR POCT B vs SC	GBP 697,730.00 (EUR 933,067.20)	GBP 31.84 (EUR 42.58)	1,001	GBP 697.32 (EUR 932.52)	0	Dominated
	AMR POCT C vs SC	GBP 683,317.00 (EUR 913,792.80)	GBP 31.18 (EUR 41.70)	864	GBP 790.97 (EUR 1,057.76)	0	Dominated
	AMR POCT D vs SC	GBP 235,532.00 (EUR 314,974.50)	GBP 10.75 (EUR 14.38)	568	GBP 414.38 (EUR 554.15)	20,676	GBP 11.39 (EUR 15.23)
	AMR POCT E vs SC	GBP 358,920.00 (EUR 479,980.00)	GBP 16.38 (EUR 21.90)	499	GBP 718.75 (EUR 961.18)	13,842	GBP 25.93 (EUR 34.68)
MSW	AMR POCT A vs SC	GBP 239,316.00 (EUR 320,034.80)	GBP 28.26 (EUR 37.79)	248	GBP 965.92 (EUR 1,291.72)	0	Dominated
	AMR POCT B vs SC	GBP 269,519.00 (EUR 360,425.00)	GBP 31.83 (EUR 42.57)	436	GBP 617.60 (EUR 825.91)	0	Dominated
	AMR POCT C vs SC	GBP 263,674.00 (EUR 352,608.50)	GBP 31.14 (EUR 41.64)	391	GBP 674.71 (EUR 902.28)	0	Dominated
	AMR POCT D vs SC	GBP 91,956.00 (EUR 122,971.80)	GBP 10.86 (EUR 14.52)	271	GBP 339.59 (EUR 454.13)	7,938	GBP 11.58 (EUR 15.49)
	AMR POCT E vs SC	GBP 132,108.00 (EUR 176,666.70)	GBP 15.60 (EUR 20.86)	248	GBP 533.21 (EUR 713.06)	5,658	GBP 23.35 (EUR 31.23)
Women	AMR POCT A vs SC	GBP 238,796.00 (EUR 319,339.40)	GBP 28.13 (EUR 37.62)	148	GBP 1,612.62 (EUR 2,156.54)	0	Dominated
	AMR POCT B vs SC	GBP 270,428.00 (EUR 361,640.60)	GBP 31.86 (EUR 42.61)	223	GBP 1,210.74 (EUR 1,619.11)	0	Dominated
	AMR POCT C vs SC	GBP 263,339.00 (EUR 352,160.50)	GBP 31.02 (EUR 41.48)	194	GBP 1,356.61 (EUR 1,814.18)	0	Dominated
	AMR POCT D vs SC	GBP 88,028.00 (EUR 117,718.90)	GBP 10.37 (EUR 13.87)	163	GBP 540.55 (EUR 722.87)	8,176	GBP 10.77 (EUR 14.40)
	AMR POCT E vs SC	GBP 110,386.00 (EUR 147,618.10)	GBP 13.00 (EUR 17.38)	148	GBP 745.45 (EUR 996.88)	6,714	GBP 16.44 (EUR 21.99)

AMR: antimicrobial resistance; MSM: men who have sex with men; MSW: men who have sex with women; POCT: point-of-care test; SC: standard care.

^a^ GBP costs were converted to EUR using a historic currency conversion of an average of 366 days from 1 July 2015 to 30 June 2016 [48]. For this time period, GBP 1 = EUR 1.34, and EUR 1 = GBP 0.75.

A strategy is ‘dominated’ if it is more expensive and provides fewer/equivalent benefits.

When avoidance of ceftriaxone use was considered, Strategy D was most cost-effective relative to SC, costing GBP 11.29 (EUR 15.09) per ceftriaxone-sparing treatment gained. These findings were consistent across all population groups.

### Sensitivity analyses

In one-way sensitivity analyses, the following four parameters had the greatest impact on cost-effectiveness per optimal treatment gained for all AMR POCT strategies and across all population groups: prevalence of azithromycin resistance; AMR POCT sensitivity; prevalence of ciprofloxacin resistance; and the cost of single detection AMR POCT. In monotherapy strategies, the cost-effectiveness model was additionally sensitive to cost of clinical management (both with and without injection), cost of ceftriaxone, and AMR POCT specificity (for strategy D). The cost multiplier for a dual detection AMR POCT impacted on AMR POCT cost-effectiveness for Strategies B and C. Tornado plots from these analyses are presented in Supplementary Figure S2.

For all strategies, variation of ICER in relation to azithromycin resistance prevalence was minimal until prevalence fell to or below 3%, at which point it increased (Supplementary Figure S3). These rises in ICER were least for strategies B and D. With the exception of strategy B where ICER were consistent for all population groups, these increases in ICER were most limited in women.

Variation in AMR POCT accuracy also showed similar patterns across all population groups. Apart from Strategy D, variation in specificity had very little effect on cost per optimal treatment gained. In contrast, as sensitivity decreased to a minimum of 90%, particularly towards the lower range, the cost per optimal treatment gained increased exponentially, except for strategy B where the relationship was linear. Strategy B also had the smallest change in ICER between maximum (100%) and minimum (90%) sensitivity (maximum difference of GBP 169.21 (EUR 226.28) per optimal treatment gained in women) compared with other strategies where the difference was in the thousands. For Strategy D, change in sensitivity had little impact on cost per optimal treatment gained, whereas when specificity decreased to below around 95.5%, the cost per optimal treatment gained started to increase exponentially. The sensitivity analyses are presented in Supplementary Figure S4.

The prevalence of ciprofloxacin resistance had very little effect on cost per optimal treatment gained in Strategies B, C and D (Supplementary Figure S5). For Strategies A and E, as ciprofloxacin resistance increased from ca 20%, there was an exponential increase in cost per optimal treatment gained for women only.

The relationship between ICER and cost of a single target AMR POCT was linear. Interestingly, as the cost of the single target AMR POCT increased, the two dual-target AMR POCTs diverged, with strategy B costing less per optimal treatment gained relative to strategy C.

For the three single target AMR POCTs (A, D and E), reducing the cost of the test had the greatest impact on cost per treatment gained. Monotherapy strategies became cost-saving (ICER < 0) for all population groups when AMR POCT cost was ≤ GBP 18 (24.07 EUR) for Strategy D, and ≤ GBP 16 (EUR 21.40) for Strategy E (Supplementary Figure S6). Strategy B had lowest costs per additional optimal treatment for dual therapy strategies.

## Discussion

We assessed the cost-effectiveness and impacts of deploying AMR POCTs for *N. gonorrhoeae.* All AMR POCT strategies assessed resulted in more optimal treatments compared with SC. Monotherapy AMR POCT strategies provided ceftriaxone-sparing options, with Strategy D reducing the use of ceftriaxone by 95%. Both outcomes are important in promoting antibiotic stewardship by minimising risks of breakthrough with ceftriaxone-resistant circulating strains, and reducing selection pressure for resistance developing to ceftriaxone, respectively.

Our cost-effectiveness analysis adapted a previously published cost-effectiveness model of introducing a dual CT/NG POCT into a SHC [28,30], and was populated using available published data, and where unavailable, using unpublished data and expert opinion. By employing a decision tree model approach we could account for sufficient complexity without over-building. However, this approach is, in contrast to using a transmission dynamic model [36], unable to assess outcomes such as the impact AMR POCTs could have on re-infection in a previously treated patient, on population prevalence or burden of disease, or on AMR evolution.

Turner et al. have adapted the same CT/NG POCT cost-effectiveness model we used for our analysis [28,30] to analyse the potential clinical and overall economic impact of an NG AMR POCT [37]. While theirs was not a cost-effectiveness analysis, and different model assumptions and parameters from ours were used, they also demonstrated that AMR POCTs could lead to overall reductions in ceftriaxone use, but that introduction of AMR POCTs incurred increased costs. Using an individual-based dynamic transmission model that incorporated partner treatment and which was applied to a London MSM population, Zienkiewicz et al. [38] also demonstrated that AMR POCTs for NG ciprofloxacin sensitivity reduced ceftriaxone use, by 70% compared with the reference scenario. An individual-based model of molecular NG AMR test use compared with culture within an NG surveillance system in remote settings found that they substantially improved the timeliness of NG AMR detection, facilitating a faster change in recommended treatment, with potential for decreasing NG AMR impact on the wider population [39]. Fingerhuth et al. [36] developed a compartmental transmission model of antibiotic-sensitive and antibiotic-resistant NG to look at proportion of resistant infections and cases averted. They showed that the clinical pathway that included an AMR POCT resulted in the lowest proportion of resistant infections after 30 years, whereas the clinical pathway with a POCT that did not test for AMR resulted in the highest. They also noted that test diagnostic performance is key for AMR POCTs to have a beneficial public health impact. The potential public health impact of AMR POCTs was confirmed by Tuite et al., with AMR POCTs delaying the proportion of isolates reaching > 5% resistance compared with empirical treatment [40]. However, it was highlighted that the AMR POCT should test for resistance to multiple anitimicrobials, otherwise non-tested, resistant, strains will be selected for. Thus, continued surveillance, including culture, should be continued. Together, these health economic and modelling evaluations highlight the possible beneficial impacts of implementing AMR POCTs on reducing ceftriaxone use and decreasing NG AMR prevalence at the population level, but the design and implementation of the tests should also be carefully considered.

As with all mathematical models, several assumptions were made (Supplementary Table S1), including AMR POCT diagnostic accuracy - a necessity as these tests are currently in early phases of development [16,41]. Future performance estimates will need to consider two elements: predictive accuracies of any biomarkers used to detect AMR; the performance of platforms and chemistries used to detect them. Variations in both may independently affect outcomes.

Our analysis had some limitations. We used the most recent NG AMR data available from GRASP at the time [29], but AMR rates constantly change and, in the sensitivity analyses, AMR prevalence alterations had the greatest impact on AMR POCT cost-effectiveness (Supplementary Figure S2). This may limit the generalisability of our results as it is not possible to know future resistance profiles. However, the results should be generalisable to the ranges used in the sensitivity analyses. In addition, as AMR POCTs are still in development, some of the model’s other epidemiological parameters will have changed by the time the AMR POCTs are available for use in routine practice, which may further limit the analyses’ applicability in the longer term. This highlights the need to continually conduct analyses such as these, to enhance our ability to predict and understand future trends. Our anlayses are also limited to data from England, with results perhaps less generalisable to other countries. This will be exacerbated by the 2019 change to 1 g ceftriaxone monotherapy, further setting it aside from guidelines in other European countries [7]. Our model also did not consider NG-positive patients coinfected with another organisms, such as CT, which would affect patient pathways and treatment options. Additional factors not considered were costs associated with treating long-term NG infection sequelae [42], costs incurred outside of the SHC, and costs or cost-savings associated with changing clinical pathways in order to accommodate the AMR POCTs. Thus the time horizon for the costs and consequences was of initial patient treatment only.

Strategy B was most effective for avoiding suboptimal treatments but the most costly to implement. Strategy D was the most cost-effective for both effectiveness outcomes (optimal treatments gained and ceftriaxone avoidance), but resulted in treatment failures, as well as nearly 15-fold higher suboptimal treatments compared with Strategy B. Both strategies B and D enabled the re-use of ciprofloxacin, previously abandoned for the treatment of NG in the UK [6].

All AMR POCT strategies were more expensive than SC, with dual therapy AMR POCT strategies more expensive than monotherapy strategies, suggesting that short-term net financial investments in AMR POCT adoption are required to gain long-term antimicrobial stewardship benefits. The O’Neill review of AMR [10] noted that accepting the initial expense of new test introduction may enable longer-term societal pay-offs by reducing infection rates and maintaining effective NG treatments. Interestingly, our sensitivity analysis suggested that even if AMR POCT costs were notably reduced, perhaps through production scale-up, dual therapy AMR POCT strategies would still not be cost-saving. However, a relatively small reduction to less than GBP 18 (EUR 24.07) per test would enable the monotherapy AMR POCT strategies to be cost-saving.

The monotherapy strategies resulted in treatment failures due to false susceptible AMR POCT results, although minimal relative to SC. Since we assumed ceftriaxone treated 100% of NG infections, there were no treatment failures for SC or dual therapy strategies. The most recent GRASP data suggest that ceftriaxone resistance remains low (no ceftriaxone resistance reported, although there is a reduction in susceptibility with 24.6% of isolates with minimum inhibitory concentrations (MICs) ≥ 0.03 mg/L in 2018 compared with 16.6% in 2017 [1]), but there are increasing concerns regarding international ceftriaxone-resistant strains [43-45]. This potentially undermines our assumption and the resulting lack of treatment failures from dual therapy AMR POCT strategies.

Most MEITRs (treatment regimen used when an earlier intended treatment regimen would have provided optimal treatment) were in Strategy C, and the least in Strategies A, B and E. Avoiding MEITRs is important because it maximises the ability to use ciprofloxacin (in Strategies A, C and E), or reduces the need for ceftriaxone use (Strategies B and D). These numbers were small compared with actual patient numbers in whom a MEITR might be used if these AMR POCTs were available more generally. For example, using national surveillance data [23,46], we estimated that over 25,000 of the 38,870 NG-diagnosed SHC patients assumed to have been treated with SC in 2015 would have had ciprofloxacin-susceptible NG. Strategies A and E would have enabled all, except 265 (Table 4), of these patients to be treated with ciprofloxacin, a 100-fold reduction in these missed opportunities.

Since a MEITR is due to susceptible infections misclassified as resistant by the AMR POCT, test specificity is key. In sensitivity analyses of AMR POCT accuracy, Strategy D was the only strategy where cost per optimal treatment gained was affected by changes in specificity. In all other strategies, cost per optimal treatment gained increased as sensitivity decreased. This is because these strategies contained an AMR POCT that included ciprofloxacin testing, so resistance (20–36%, dependent on population group [29]) was detected and optimal treatment could be given. In contrast, if AMR POCT sensitivity in these strategies fell, true ciprofloxacin-resistant cases were missed and the patient suboptimally treated. Strategy D, where the AMR POCT was for azithromycin only, was the only strategy where ciprofloxacin was given without resistance-testing - as the specificity decreased, more patients received false-positive azithromycin resistance results and were treated with ciprofloxacin. Due to high ciprofloxacin resistance prevalence, this treatment was suboptimal in a large number of cases. Following the logic of the other strategies, if azithromyin resistance prevalence increased, cost per optimal treatment gained in Strategy D would become sensitive to both AMR POCT specifity and sensitivity.

Thus, prevalence of resistance has important implications for AMR POCT accuracy requirements and ICER of optimal treatments gained. In the azithromycin resistance sensitivity analyses, ICER increased when resistance fell below ca 3% (well below current UK azithromycin resistance prevalence, reported at ca 9.7% [1]), primarily because when azithromycin resistance is low, there is little value in testing for it (Strategies B, C and D) and there will be few treatment failures from background resistance (Strategies A and E). In the ciprofloxacin resistance sensitivity analysis, an effect on ICER was only seen in women in strategies A and E (because of lower baseline ciprofloxacin resistance prevalence).

From a population-level antimicrobial stewardship public health perspective, increasing the number of suboptimal treatments may eventually lead to an increased number of resistant infections [36]. The relative public health importance of a smaller total number of suboptimal treatments with a few treatment failures vs a higher number of suboptimal treatments with no failures warrants further investigation, and could be included in future transmission model analyses. Furthermore, the long-term public health impact of preserving ceftriaxone use while increasing the risk of treatment failures from monotherapy strategies (vs maintaining ceftriaxone in the earlier intended treatment regimen with an increase in suboptimal treatments and no adequate treatment alternative), should also be investigated.

## Conclusion

Once developed, AMR POCTs could have wide-ranging implications for clinical decisionmaking globally, including the potential re-use of antibiotics previously abandoned for the treatment of NG, ensuring the right treatment is given to the right person at the right time (precision medicine). Although it may be necessary to accept net health system investment to enable longer-term societal pay-offs by reducing infection rates and maintaining effective NG treatments, a relatively small reduction in test cost could enable some AMR POCT strategies to be cost-saving.

empirical study and modelling is required to optimise their use for public health benefit.

## Supplementary Data

2014000 Supplementary Figures

## Supplementary Data

237000 Supplementary Tables
