# Supplementary material for: Antimicrobial resistance point-of-care testing for gonorrhoea treatment regimens: cost-effectiveness and impact on ceftriaxone use of five hypothetical strategies compared with standard care in England sexual health clinics
Source: Euro Surveill. 2020 Oct 29;25(43):1900402. doi: 10.2807/1560-7917.ES.2020.25.43.1900402 (PMC7596918; doi:10.2807/1560-7917.ES.2020.25.43.1900402)
Supplement: Supplementary Figures [file 1900402_SADIQ_SupplementaryFigures.pdf]

Supplementary Figure S1. Antimicrobial resistance point-of-care test (AMR-POCT) patient pathways

This supplementary material is hosted by Eurosurveillance as supporting information alongside the article “Antimicrobial resistance point-of-care testing for gonorrhoea treatment regimens: cost-effectiveness and impact on ceftriaxone use of five hypothetical strategies compared with standard care in England sexual health clinics” on behalf of the authors who remain responsible for the accuracy and appropriateness of the content. The same standards for ethics, copyright, attributions and permissions as for the article apply. Eurosurveillance is not responsible for the maintenance of any links or email addresses provided therein.

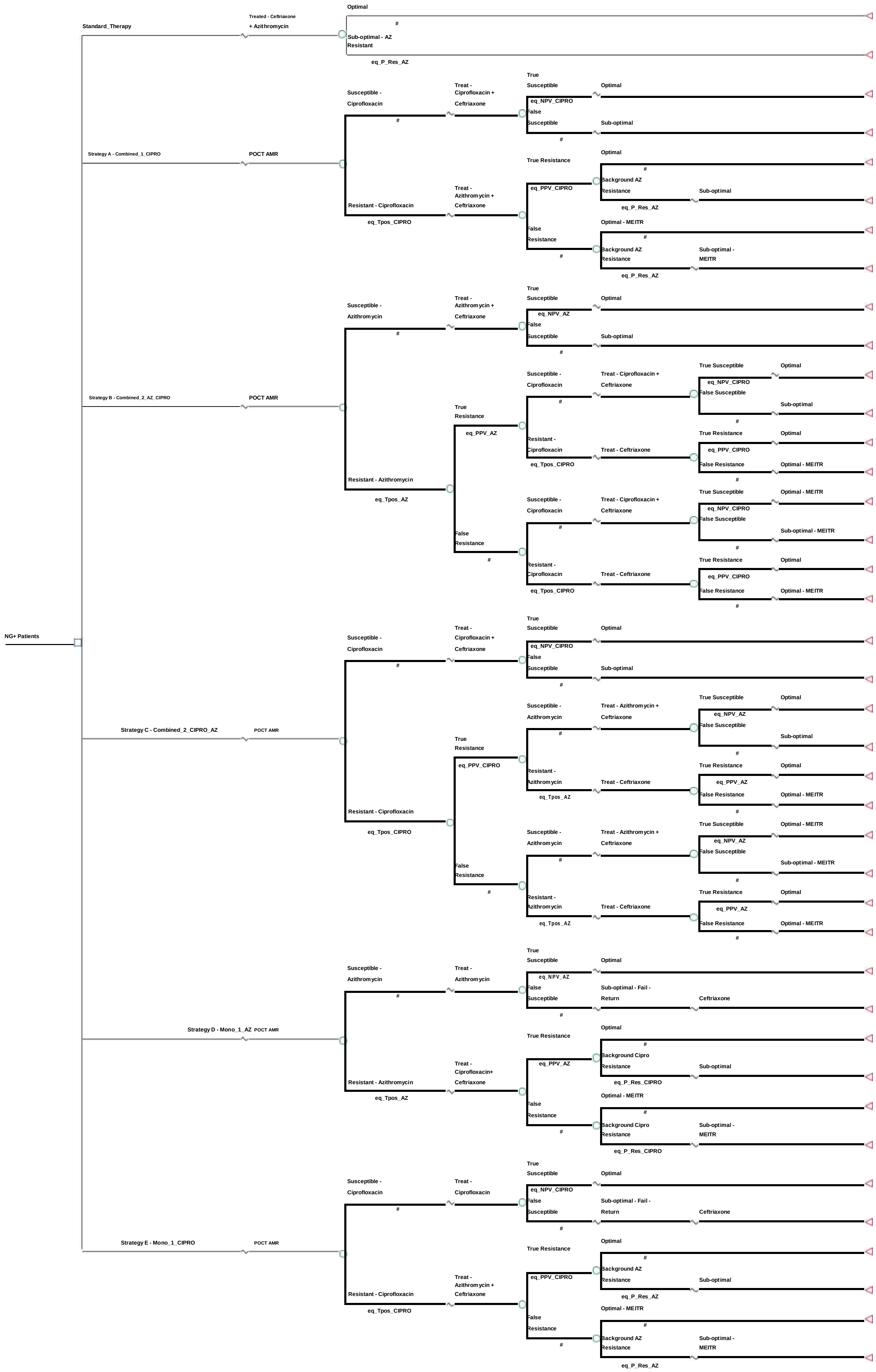
NG+ Patients
Standard_Therapy
Treated - Ceftriaxone
+ Azithromycin
Optimal
#
Sub-optimal - AZ
Resistant
eq_P_Res_AZ
Strategy A - Combined_1_CIPRO
POCT AMR
Susceptible -
Ciprofloxacin
#
Treat -
Ciprofloxacin +
Ceftriaxone
True
Susceptible
eq_NPV_CIPRO
False
Susceptible
#
Optimal
Sub-optimal
Resistant - Ciprofloxacin
eq_Tpos_CIPRO
Treat -
Azithromycin +
Ceftriaxone
True Resistance
eq_PPV_CIPRO
False
Resistance
#
Optimal
#
Background AZ
Resistance
Sub-optimal
eq_P_Res_AZ
Optimal - MEITR
#
Background AZ
Resistance
Sub-optimal -
MEITR
eq_P_Res_AZ
Strategy B - Combined_2_AZ_CIPRO
POCT AMR
Susceptible -
Azithromycin
#
Treat -
Azithromycin +
Ceftriaxone
True
Susceptible
eq_NPV_AZ
False
Susceptible
#
Optimal
Sub-optimal
Resistant - Azithromycin
eq_Tpos_AZ
True
Resistance
eq_PPV_AZ
Susceptible -
Ciprofloxacin
#
Treat - Ciprofloxacin +
Ceftriaxone
True Susceptible
eq_NPV_CIPRO
False Susceptible
#
Optimal
Sub-optimal
Resistant -
Ciprofloxacin
eq_Tpos_CIPRO
Treat - Ceftriaxone
True Resistance
eq_PPV_CIPRO
False Resistance
#
Optimal
Optimal - MEITR
False
Resistance
#
Susceptible -
Ciprofloxacin
#
Treat - Ciprofloxacin +
Ceftriaxone
True Susceptible
eq_NPV_CIPRO
False Susceptible
#
Optimal - MEITR
Sub-optimal - MEITR
Resistant -
Ciprofloxacin
eq_Tpos_CIPRO
Treat - Ceftriaxone
True Resistance
eq_PPV_CIPRO
False Resistance
#
Optimal
Optimal - MEITR
Strategy C - Combined_2_CIPRO_AZ
POCT AMR
Susceptible -
Ciprofloxacin
#
Treat -
Ciprofloxacin +
Ceftriaxone
True
Susceptible
eq_NPV_CIPRO
False
Susceptible
#
Optimal
Sub-optimal
Resistant - Ciprofloxacin
eq_Tpos_CIPRO
True
Resistance
eq_PPV_CIPRO
Susceptible -
Azithromycin
#
Treat - Azithromycin +
Ceftriaxone
True Susceptible
eq_NPV_AZ
False Susceptible
#
Optimal
Sub-optimal
Resistant -
Azithromycin
eq_Tpos_AZ
Treat - Ceftriaxone
True Resistance
eq_PPV_AZ
False Resistance
#
Optimal
Optimal - MEITR
False
Resistance
#
Susceptible -
Azithromycin
#
Treat - Azithromycin +
Ceftriaxone
True Susceptible
eq_NPV_AZ
False Susceptible
#
Optimal - MEITR
Sub-optimal - MEITR
Resistant -
Azithromycin
eq_Tpos_AZ
Treat - Ceftriaxone
True Resistance
eq_PPV_AZ
False Resistance
#
Optimal
Optimal - MEITR
Strategy D - Mono_1_AZ
POCT AMR
Susceptible -
Azithromycin
#
Treat -
Azithromycin
True
Susceptible
eq_NPV_AZ
False
Susceptible
#
Optimal
Sub-optimal - Fail -
Return
Ceftriaxone
Resistant - Azithromycin
eq_Tpos_AZ
Treat -
Ciprofloxacin+
Ceftriaxone
True Resistance
eq_PPV_AZ
False
Resistance
#
Optimal
#
Background Cipro
Resistance
Sub-optimal
eq_P_Res_CIPRO
Optimal - MEITR
#
Background Cipro
Resistance
Sub-optimal -
MEITR
eq_P_Res_CIPRO
Strategy E - Mono_1_CIPRO
POCT AMR
Susceptible -
Ciprofloxacin
#
Treat -
Ciprofloxacin
True
Susceptible
eq_NPV_CIPRO
False
Susceptible
#
Optimal
Sub-optimal - Fail -
Return
Ceftriaxone
Resistant - Ciprofloxacin
eq_Tpos_CIPRO
Treat -
Azithromycin +
Ceftriaxone
True Resistance
eq_PPV_CIPRO
False
Resistance
#
Optimal
#
Background AZ
Resistance
Sub-optimal
eq_P_Res_AZ
Optimal - MEITR
#
Background AZ
Resistance
Sub-optimal -
MEITR
eq_P_Res_AZ

Standard Care (SC)
Standard care with dual-therapy of intramuscular ceftriaxone (500mg) and oral azithromycin (1g single dose).

Dual-therapy, including ceftriaxone
A) AMR-POCT for ciprofloxacin resistance only; infections identified as not resistant to ciprofloxacin are given oral ciprofloxacin (500mg) plus ceftriaxone (500mg). Infections identified as ciprofloxacin resistant are given SC.
B) Dual AMR-POCT for azithromycin and ciprofloxacin resistance; if no azithromycin resistance is identified, SC is given. If azithromycin resistant, ciprofloxacin (500mg) and ceftriaxone (500mg) are given unless there is ciprofloxacin resistance, in which case ceftriaxone (500mg) is given alone.
C) Dual AMR-POCT for ciprofloxacin and azithromycin resistance; if no ciprofloxacin resistance is identified, ciprofloxacin (500mg) and ceftriaxone (500mg) are given. If ciprofloxacin resistant, SC is given, unless there is also azithromycin resistance, when ceftriaxone (500mg) is given alone.

Monotherapy optimisation
D) AMR-POCT for azithromycin resistance: if no azithromycin resistance is identified, azithromycin (2g) is given. If azithromycin resistant, ceftriaxone (500mg) and ciprofloxacin (500mg) dual-therapy is given. If the AMR-POCT incorrectly shows no resistance (false negative for AMR), it is assumed the treatment fails. The treatment failure would be identified in the test-of-cure (TOC) and the patient would then receive 500mg ceftriaxone.
E) AMR-POCT for ciprofloxacin; if no ciprofloxacin resistance is identified, 500mg ciprofloxacin monotherapy is given. If ciprofloxacin resistant, SC is given. If the AMR-POCT incorrectly shows no resistance, monotherapy is assumed to fail, the patient returns and receives 500mg ceftriaxone alone.

Supplementary Figure S2. Sensitivity analysis tornado plots by strategy and population group

This supplementary material is hosted by Eurosurveillance as supporting information alongside the article "Antimicrobial resistance point-of-care testing for gonorrhoea treatment regimens: cost-effectiveness and impact on ceftriaxone use of five hypothetical strategies compared with standard care in England sexual health clinics" on behalf of the authors who remain responsible for the accuracy and appropriateness of the content. The same standards for ethics, copyright, attributions and permissions as for the article apply. Eurosurveillance is not responsible for the maintenance of any links or email addresses provided therein.

<table>
<tr><th>Strategy</th><th>MSM</th><th>MSW</th><th>Women</th></tr>
<tr><td>A</td><td>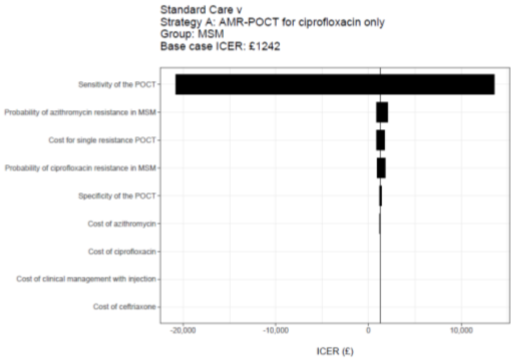
</td><td>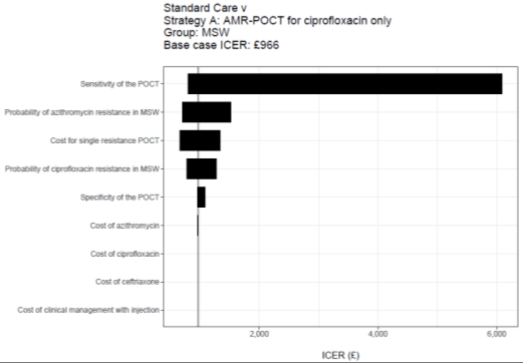
</td><td>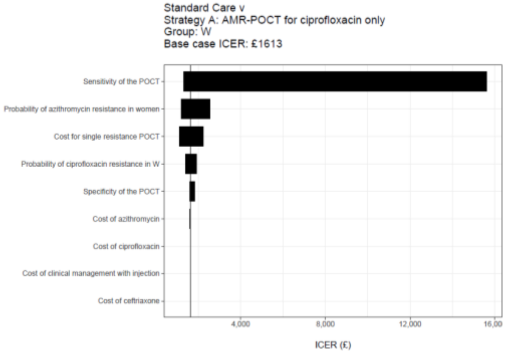
</td></tr>
<tr><td>B</td><td>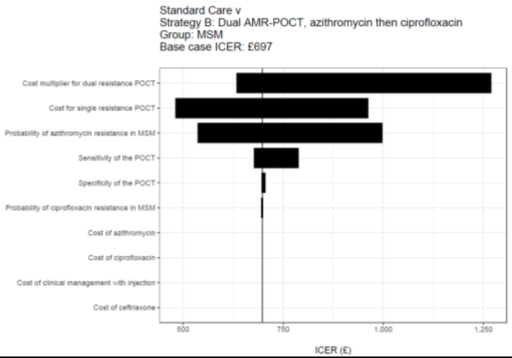
</td><td>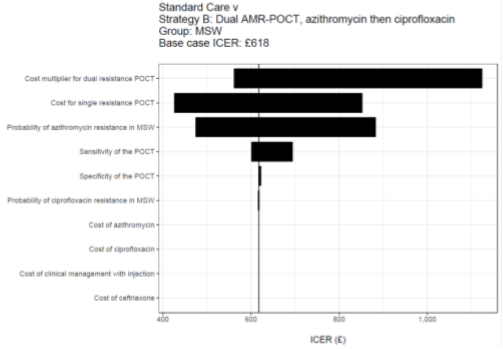
</td><td>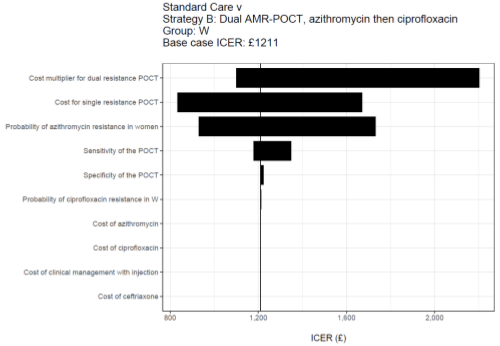
</td></tr>
</table>

C

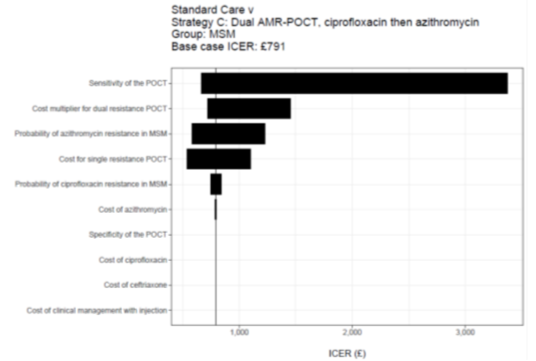

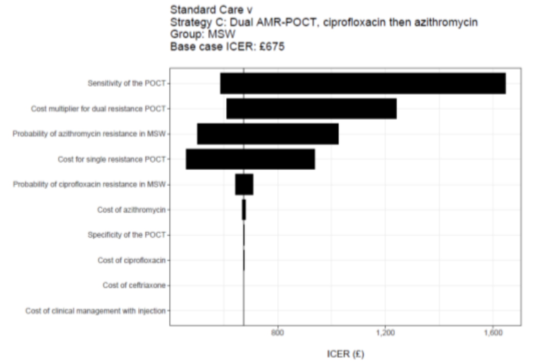

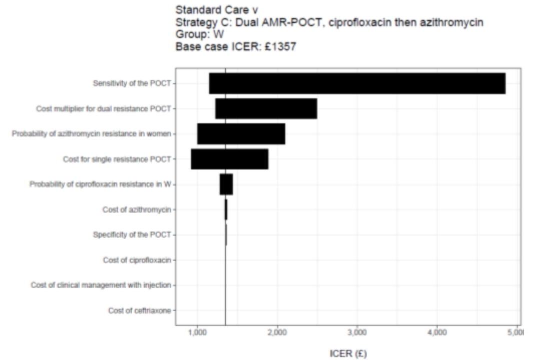

D

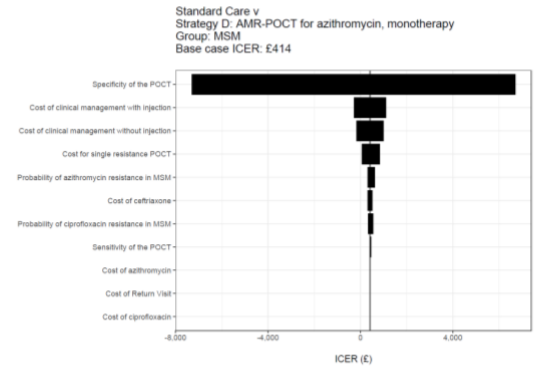

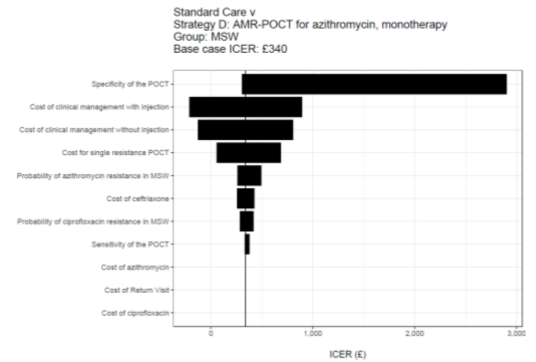

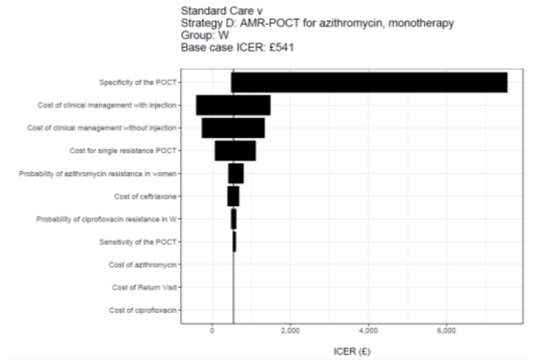

E

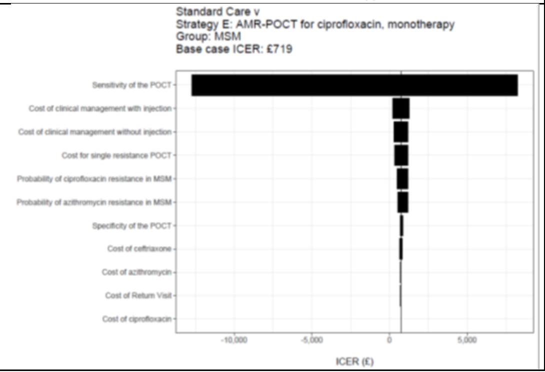

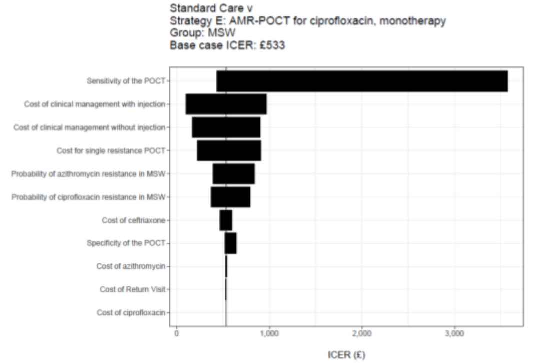

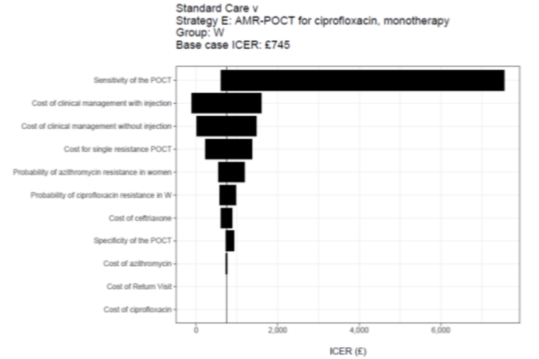

AMR, antimicrobial resistance; POCT, point-of-care test; ICER, incremental cost-effectiveness ratio; MSM, men-who-have-sex-with-men; MSW, men-who-have-sex-with-women; W, women

Supplementary Figure S3. Sensitivity analysis demonstrating the changes in incremental cost effectiveness ratios (ICERs) with varying prevalence of azithromycin resistant NG

This supplementary material is hosted by Eurosurveillance as supporting information alongside the article "Antimicrobial resistance point-of-care testing for gonorrhoea treatment regimens: cost-effectiveness and impact on ceftriaxone use of five hypothetical strategies compared with standard care in England sexual health clinics" on behalf of the authors who remain responsible for the accuracy and appropriateness of the content. The same standards for ethics, copyright, attributions and permissions as for the article apply. Eurosurveillance is not responsible for the maintenance of any links or email addresses provided therein.

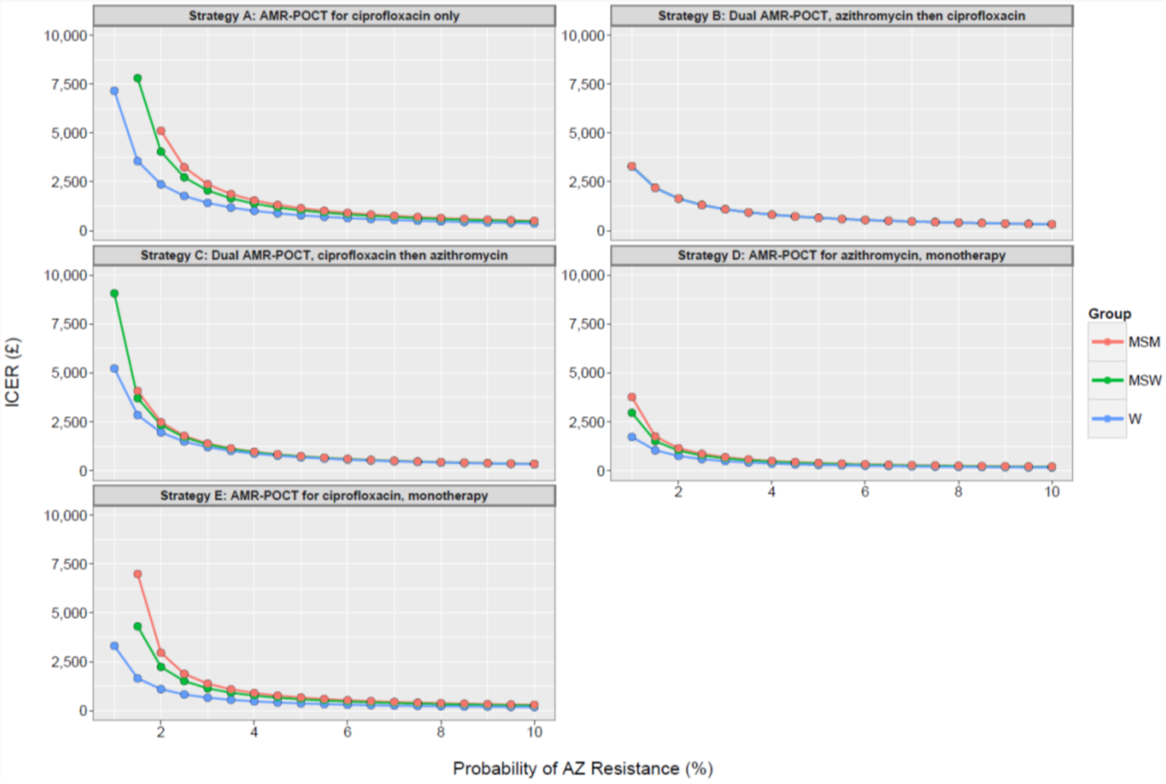

The five graphs represent the five different NG AMR-POCT strategies, A-E (see text). MSM: men-who-have-sex-with-men; MSW: men-who-have-sex-with-women; W: women; AMR, antimicrobial resistance; POCT, point-of-care test; ICER, incremental cost-effectiveness ratio

To enable detail at the lower values to be seen, any data points >10,000 ICER have not been included on the graph.

Supplementary Figure S4. AMR-POCT sensitivity and specificity sensitivity analyses by strategy and by population group

This supplementary material is hosted by Eurosurveillance as supporting information alongside the article "Antimicrobial resistance point-of-care testing for gonorrhoea treatment regimens: cost-effectiveness and impact on ceftriaxone use of five hypothetical strategies compared with standard care in England sexual health clinics" on behalf of the authors who remain responsible for the accuracy and appropriateness of the content. The same standards for ethics, copyright, attributions and permissions as for the article apply. Eurosurveillance is not responsible for the maintenance of any links or email addresses provided therein.

<table>
<tr><th>Strategy</th><th>MSM</th><th>MSW</th><th>Women</th></tr>
<tr><td>A</td><td>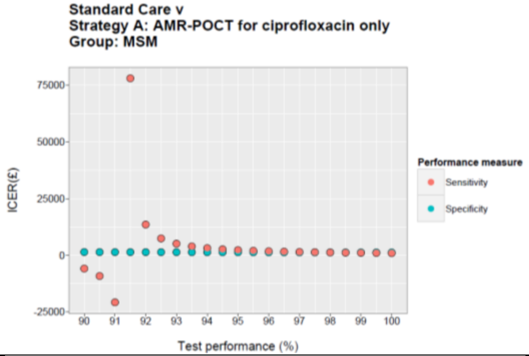
</td><td>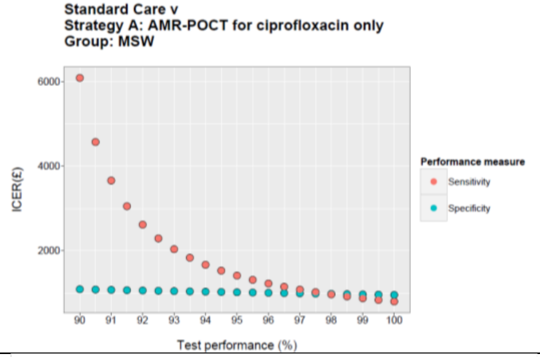
</td><td>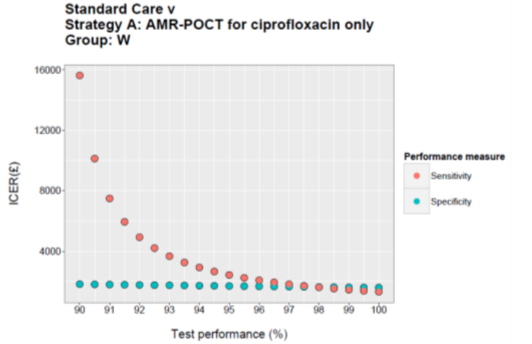
</td></tr>
<tr><td>B</td><td>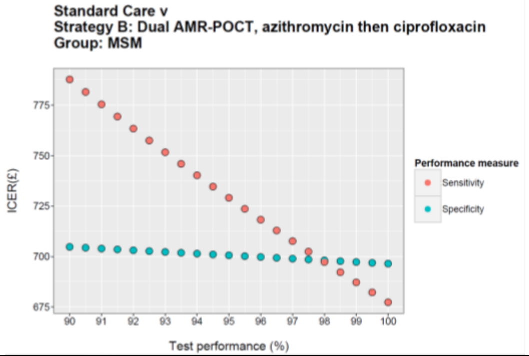
</td><td>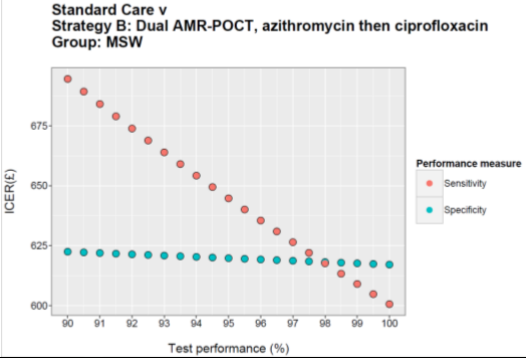
</td><td>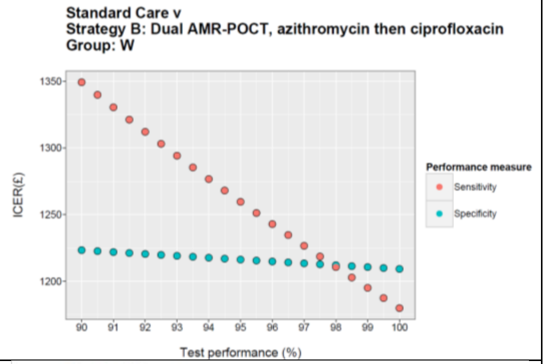
</td></tr>
<tr><td>C</td><td>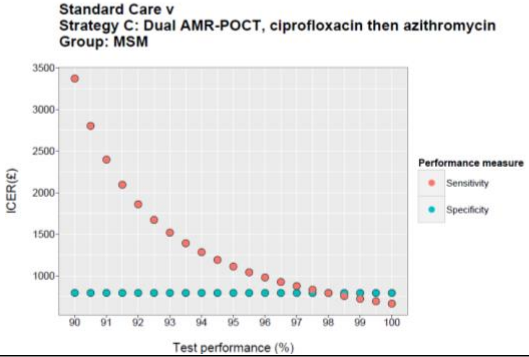
</td><td>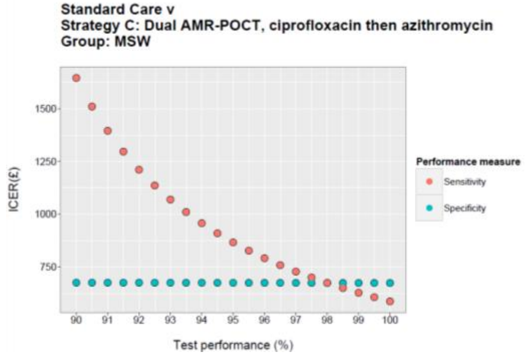
</td><td>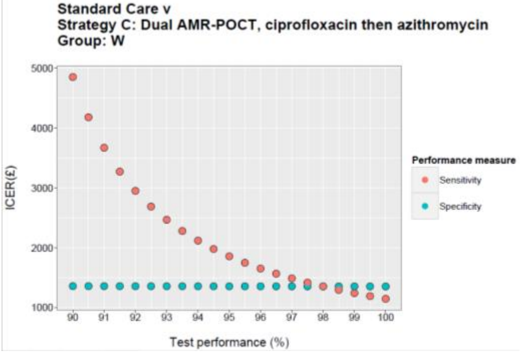
</td></tr>
</table>

D

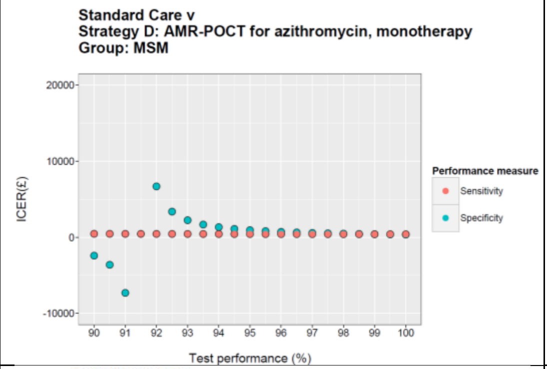

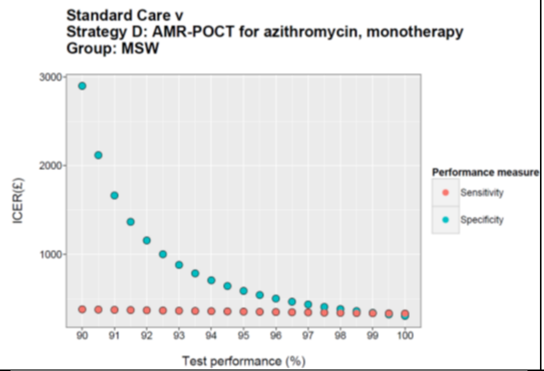

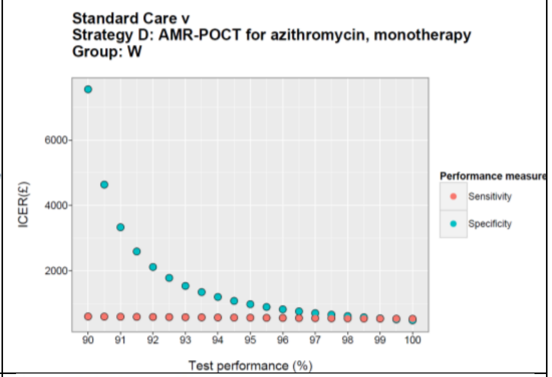

E

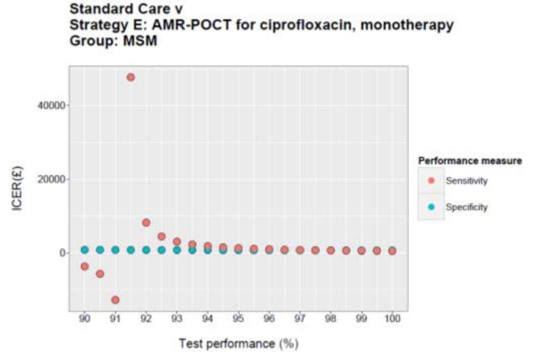

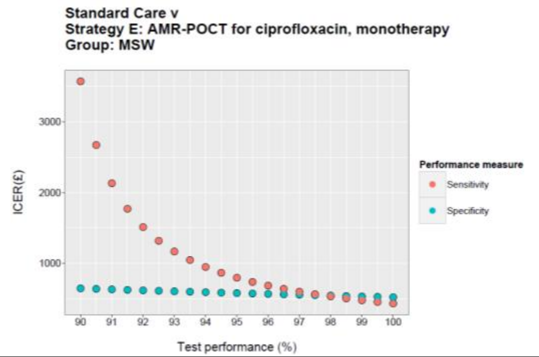

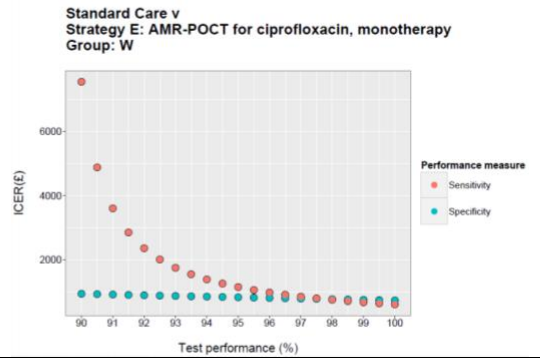

AMR, antimicrobial resistance; POCT, point-of-care test; ICER, incremental cost-effectiveness ratio; MSM: men-who-have-sex-with-men; MSW: men-who-have-sex-with-women; W: women

Supplementary Figure S5. Sensitivity analysis demonstrating the changes in incremental cost effectiveness ratios (ICERs) with varying prevalence of ciprofloxacin resistant NG

This supplementary material is hosted by Eurosurveillance as supporting information alongside the article "Antimicrobial resistance point-of-care testing for gonorrhoea treatment regimens: cost-effectiveness and impact on ceftriaxone use of five hypothetical strategies compared with standard care in England sexual health clinics" on behalf of the authors who remain responsible for the accuracy and appropriateness of the content. The same standards for ethics, copyright, attributions and permissions as for the article apply. Eurosurveillance is not responsible for the maintenance of any links or email addresses provided therein.

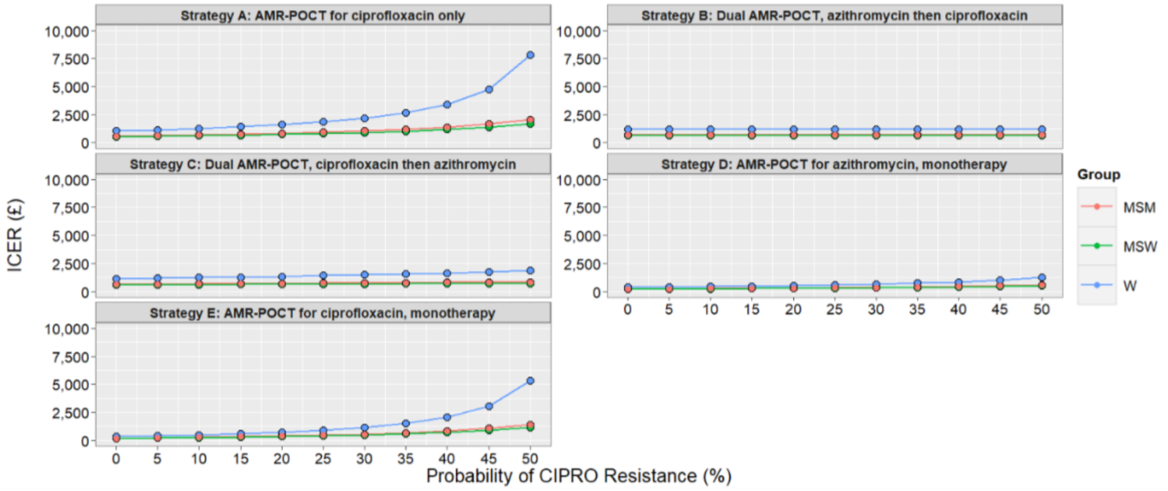

The five graphs represent the five different NG AMR-POCT strategies, A-E (see text).
AMR, antimicrobial resistance; POCT, point-of-care test; ICER, incremental cost-effectiveness ratio; MSM: men-who-have-sex-with-men; MSW: men-who-have-sex-with-women; W: women

Supplementary Figure S6. Cost of single-target AMR-POCT sensitivity analyses by population group

This supplementary material is hosted by Eurosurveillance as supporting information alongside the article “Antimicrobial resistance point-of-care testing for gonorrhoea treatment regimens: cost-effectiveness and impact on ceftriaxone use of five hypothetical strategies compared with standard care in England sexual health clinics” on behalf of the authors who remain responsible for the accuracy and appropriateness of the content. The same standards for ethics, copyright, attributions and permissions as for the article apply. Eurosurveillance is not responsible for the maintenance of any links or email addresses provided therein.

MSM	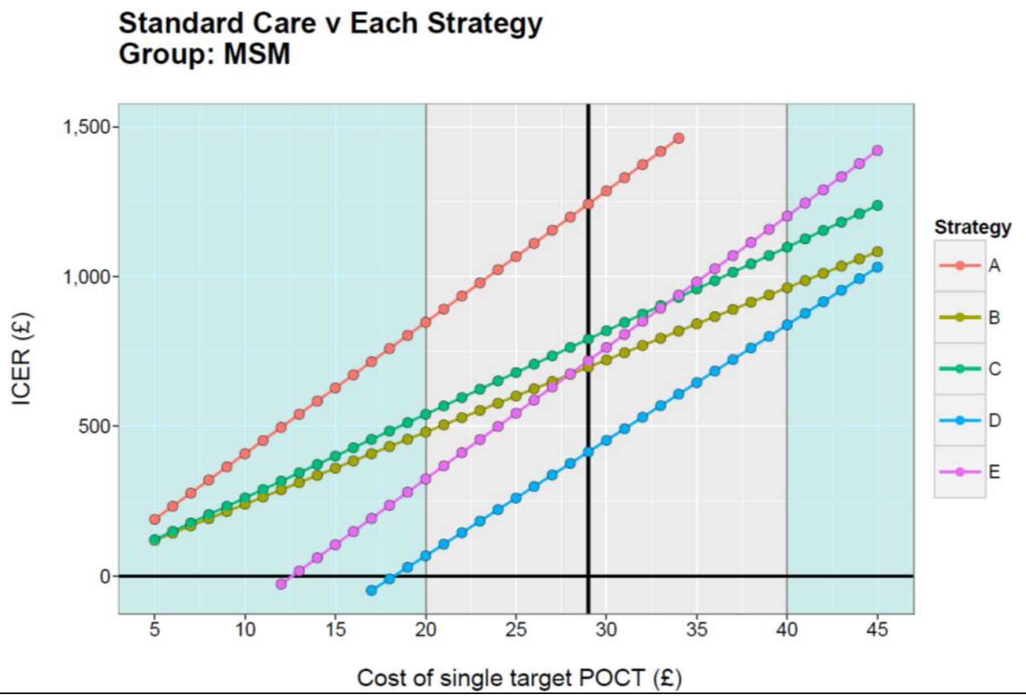

MSW	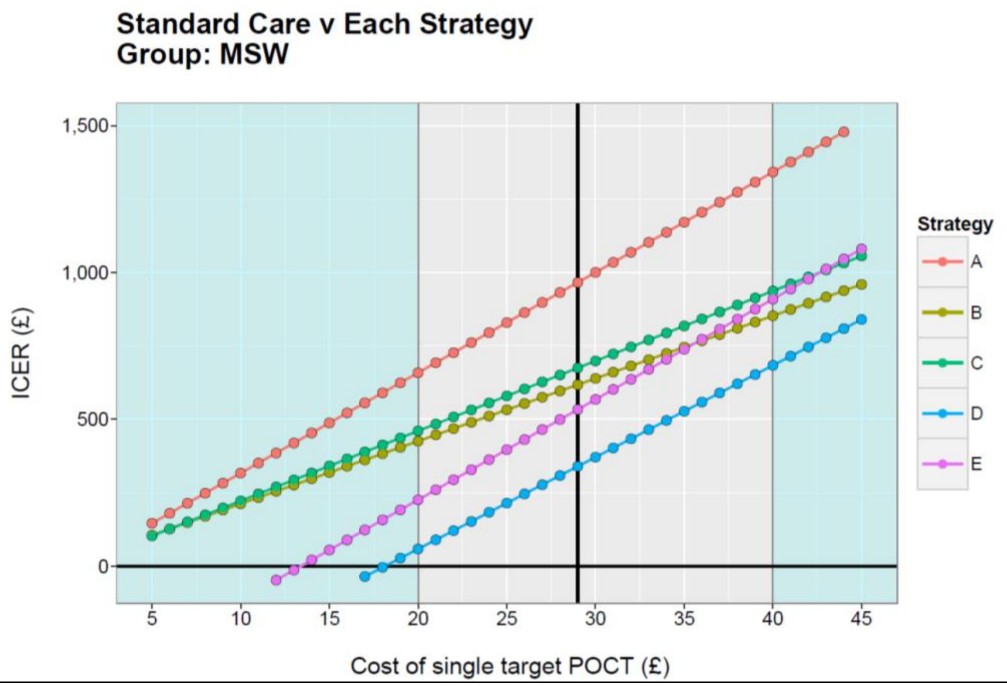Standard Care v Each Strategy<br>Group: MSW<br>ICER (£)<br>1,500<br>1,000<br>500<br>0<br>5<br>10<br>15<br>20<br>25<br>30<br>35<br>40<br>45<br>Cost of single target POCT (£)<br>Strategy<br>A<br>B<br>C<br>D<br>E

<table>
<tr>
<td>Women</td>
<td>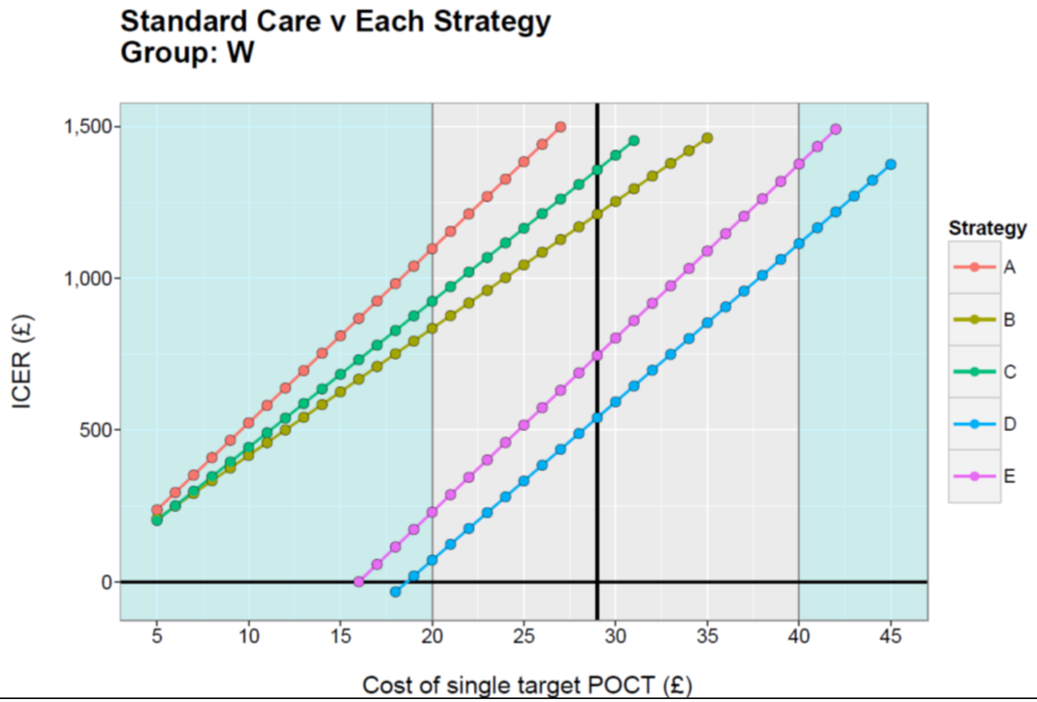
</td>
</tr>
</table>

AMR, antimicrobial resistance; POCT, point-of-care test; ICER, incremental cost-effectiveness ratio; MSM: men-who-have-sex-with-men; MSW: men-who-have-sex-with-women; W: women.
Strategies B and C are dual-target AMR-POCTs; their cost is that of a single target AMR-POCT, with a 10% multiplier (i.e. the base-case for AMR-pPOCT strategies B and C is £31.90).
