# Supplementary material for: Antimicrobial resistance point-of-care testing for gonorrhoea treatment regimens: cost-effectiveness and impact on ceftriaxone use of five hypothetical strategies compared with standard care in England sexual health clinics
Source: Euro Surveill. 2020 Oct 29;25(43):1900402. doi: 10.2807/1560-7917.ES.2020.25.43.1900402 (PMC7596918; doi:10.2807/1560-7917.ES.2020.25.43.1900402)
Supplement: Supplementary Tables [file 1900402_SADIQ_SupplementaryTables.pdf]

Supplementary Table S1. NG model assumptions

This supplementary material is hosted by Eurosurveillance as supporting information alongside the article “Antimicrobial resistance point-of-care testing for gonorrhoea treatment regimens: cost-effectiveness and impact on ceftriaxone use of five hypothetical strategies compared with standard care in England sexual health clinics” on behalf of the authors who remain responsible for the accuracy and appropriateness of the content. The same standards for ethics, copyright, attributions and permissions as for the article apply. Eurosurveillance is not responsible for the maintenance of any links or email addresses provided therein.

**NG model assumptions/parameters**	
**Assumptions**	
1	All patients entering the model are true positives for NG
2	AMR-POCT conducted in SHC
3	Dual AMR-POCTs results available simultaneously
4	No presumptive treatment
5	Mathematical independence between the different resistant strains of NG
6	Performance characteristics comparable to other NAAT rapid/POC tests
7	30-minute test run-time
8	100% adherence to AMR-POCT
9	Single dose therapy for all treatments
10	100% adherence to the antibiotics prescribed
11	Patients with monotherapy treatment failure would return
12	No attrition (loss to follow-up)
13	Treatment with ceftriaxone (whether given as monotherapy or in combination for dual therapy) is an effective cure for 100% of NG infections
14	No variation in treatment regimens by population group (females, MSW, MSM)
15	All NG positive samples still sent to laboratory for culture and phenotypic resistance testing
**Parameters not accounted for/included:**	
1	Complications associated with STI infections, e.g. PID
2	Patient side-effects or allergies to medications (e.g. penicillin)
3	Co-infections
4	Costs incurred beyond the SHC (health provider)

5	Initial cost of diagnosing NG
6	Cost of implementing a change into clinical practice

Supplementary Table S2. Model definitions

This supplementary material is hosted by Eurosurveillance as supporting information alongside the article “Antimicrobial resistance point-of-care testing for gonorrhoea treatment regimens: cost-effectiveness and impact on ceftriaxone use of five hypothetical strategies compared with standard care in England sexual health clinics” on behalf of the authors who remain responsible for the accuracy and appropriateness of the content. The same standards for ethics, copyright, attributions and permissions as for the article apply. Eurosurveillance is not responsible for the maintenance of any links or email addresses provided therein.

Term	Definition
Positive test<br>*P(T+)*	Denotes that the test has classified the sample as resistant to a specific antibiotic or class of antibiotics
Negative test<br>*P(T-)*	Denotes that the test has classified the sample as susceptible to a specific antibiotic or class of antibiotics
Positive predictive value<br>*P(R+|T+)*	Denotes that the probability of a resistant infection conditional upon a positive test
Negative predictive value<br>*P(R-|T-)*	Denotes the probability of a susceptible infection conditional upon a negative test
Optimal treatment regimen	1) Treatment regimen cures NG<br>2) NG is not resistant to any of the antibiotics given<br>3) An earlier intended treatment regimen was used, or a later intended treatment regimen was used because earlier intended treatment regimens contained antibiotics to which NG was resistant
Missed earlier intended treatment regimen (MEITR)[a]	1) Treatment regimen cures NG<br>2) NG is not resistant to any of the antibiotics given<br>3) A treatment regimen was used when an earlier intended treatment regimen would have provided optimal treatment
Sub-optimal treatment regimen	1) Treatment regimen cures NG in dual therapy strategies; treatment regimen failure in monotherapy strategies<br>2) Treatment regimen which contains antibiotics against which there is NG resistance
Sub-optimal treatment regimen – MEITR[b]	1) Treatment cures NG in dual therapy strategies; treatment failure in monotherapy strategies<br>2) Treatment regimen which contains antibiotics against which there is NG resistance<br>3) An earlier intended treatment regimen was misclassified as containing NG-resistant antibiotics and if used in place of the selected treatment would have made the combination an optimal treatment

Treatment failure	1) Treatment regimen fails to cure NG infection due to resistance to an antibiotic given as monotherapy

[a] This will occur when a patient tests positive for resistance but the test was incorrect

[b] This will occur when a patient tests positive for resistance, the test was incorrect, and the treatment given contained drugs against which there was NG resistance

Supplementary Table S3. Breakdown of cost data

This supplementary material is hosted by Eurosurveillance as supporting information alongside the article “Antimicrobial resistance point-of-care testing for gonorrhoea treatment regimens: cost-effectiveness and impact on ceftriaxone use of five hypothetical strategies compared with standard care in England sexual health clinics” on behalf of the authors who remain responsible for the accuracy and appropriateness of the content. The same standards for ethics, copyright, attributions and permissions as for the article apply. Eurosurveillance is not responsible for the maintenance of any links or email addresses provided therein.

**Cost input**		**Cost**	
Management of NG - oral medication excludes cost of POCT and drugs	**Staff grade [time]**	**GBP**	**Euros**
	Admin/Clerical [1 min]	0.53	0.71
	Doctor 7/8 blend [16 mins]	23.89	31.95
	Nurse 5/6/7/8 blend [10 mins]	9.33	12.48
	Health Adviser blend [6 mins[1]]	6.26	8.37
	**Consumables**		
	Examination and tests	3.00	4.01
	Health promotion and partner notification	2.04	2.73
	**Pathology**		
	Lab processing of NG	7.95	10.63
	**Total**	53.00	70.88
Management of NG - IM injection excludes cost of POCT and drugs	**Staff grade [time]**		
	Nurse 5/6/7/8 blend [30 mins]	27.99	37.43
	Doctor 7/8 blend [10 mins]	14.93	19.977
	Health Adviser blend [6 mins[a]]	6.26	8.37
	Admin/clerical [1 min]	0.53	0.71
	**Consumables**		
	Examination and tests	3.00	4.01
	Injection	0.03	0.04
	Health promotion and partner notification	2.04	2.73
	**Pathology**		
	Lab processing of NG	7.95	10.63
	**Total**	62.74	83.90
Additional cost of performing AMR POCT	**Staff grade [time]**		
	Nurse 5/6/7/8 blend [4 mins]	3.74	5.00
	Admin/clerical [2 mins]	1.07	1.43
	**Total**	4.81	6.43
Test of cure for NG (includes cost of POCT for NG)	**Staff grade [time]**		
	Nurse 5/6/7/8 blend [10 mins]	9.33	12.48
	Health Adviser blend [5 mins]	5.22	6.98
	Admin/clerical [5 mins]	2.67	3.57
	**Consumables**		
	Sample collection	3.80	5.08

	**Pathology**		
	POCT	24.00	32.09
	Lab processing of NG	7.95	10.63
	**Total**	52.97	70.84
Return visit due to treatment failure excludes cost of drugs does not include any further pathology cost	**Staff grade [time]**		
	Admin/clerical [5 mins]	2.67	3.57
	Nurse 5/6/7/8 blend [30 mins]	27.99	37.43
	Doctor 7/8 blend [10 mins]	14.93	19.97
	**Consumables**		
	Examination	0.83	1.11
	Health promotion	1.59	2.13
	**Total**	48.01	64.20

Costs are adapted from a previous microcosting model. Adams, 2014 [30] and were inflated to 2015/16 costs using the Hospital and Community Health Services (HCHS) Inflation Indices 2015 produced by the Personal Social Services Research Unit [34]. No data were available for inflation from 2014/15 to 2015/16 so it was assumed to the same as between 2013/2014 and 2014/15. The UK hospital consumer price index for health services shows similar annual growth in this sector from 2014 (93.2 in 2013, 97.1 in 2014 and 100 in 2015), which validates this assumption [35]. GBP costs were converted to Euros using a historic currency conversion of an average of 366 days from the 1st July 2015 to 30th June January 2016 [33]. For this time period, 1 GBP = 1.34 Euros, and 1 Euro = 0.75 GBP.

[a]20% of patients receive 30 minutes Health Adviser time

Supplementary Table S4. Total costs, treatments used, and treatment outcomes for SC and AMR-POCT strategies; MSM (n=21,915)

This supplementary material is hosted by Eurosurveillance as supporting information alongside the article "Antimicrobial resistance point-of-care testing for gonorrhoea treatment regimens: cost-effectiveness and impact on ceftriaxone use of five hypothetical strategies compared with standard care in England sexual health clinics" on behalf of the authors who remain responsible for the accuracy and appropriateness of the content. The same standards for ethics, copyright, attributions and permissions as for the article apply. Eurosurveillance is not responsible for the maintenance of any links or email addresses provided therein.

Strategy	Total cost[a]	Number of antibiotics used to treat NG			Number of optimal treatments[b]	Number of sub-optimal treatments[c]	Number of MEITR[d]	Number of treatment failures[e]
		Ceftriaxone	Azithromycin	Ciprofloxacin				
Standard care	£1,610,314<br>2,153,456 EUR	21,915	21,915	0	20,885	1,030	N/A	0
A) Dual therapy single POCT - Ciprofloxacin	£2,230,589<br>2,982,944 EUR	21,915	7,914	14,001	21,384	531	140	0
B) Dual therapy dual POCT - Azithromycin Ciprofloxacin	£2,308,044<br>3,086,524 EUR	21,915	20,697	778	21,886	29	141	0
C) Dual therapy dual POCT - Ciprofloxacin Azithromycin	£2,293,632<br>3,067,251 EUR	21,915	7,474	14,001	21,749	166	566	0

D) Monotherapy Azithromycin	£1,845,847 2,468,432 EUR	1,239	20,697	1,218	21,453	462	209	21
E) Monotherapy Ciprofloxacin	£1,969,234 2,633,436 EUR	8,073	7,914	14,001	21,384	531	140	159

SC, Standard Care; AMR, antimicrobial resistance; POCT, point-of-care test; NG, *Neisseria gonorrhoeae*; MEITR, missed earlier intended treatment regimen; MSM, men who have sex with men; N/A, Not Applicable

[a] GBP costs were converted to Euros using a historic currency conversion of an average of 366 days from the 1st July 2015 to 30th June January 2016 [33]. For this time period, 1 GBP = 1.34 Euros, and 1 Euro = 0.75 GBP.
[b]'optimal' refers to a treatment regimen which cures the NG infection and does not contain any antibiotic against which there is resistance
[c]'sub-optimal' refers to a treatment regimen which contains antibiotics against which there is NG resistance - if the treatment is a monotherapy it will result in treatment failure
[d]'missed earlier intended treatment regimen' (MEITR) refers to a treatment regimen which cures the NG infection and does not contain any antibiotic against which there is resistance, but a treatment regimen was used when an earlier intended treatment regimen would have provided optimal treatment – a MEITR is due to a false-resistant AMR-POCT result
[e]'treatment failure' refers to failure to cure an NG infection due to resistance to an antibiotic given as monotherapy and is due to a false-susceptible AMR-POCT result

Supplementary Table S5. Total costs, treatments used, and treatment outcomes for SC and AMR-POCT strategies; Women (n=8,488)

This supplementary material is hosted by Eurosurveillance as supporting information alongside the article "Antimicrobial resistance point-of-care testing for gonorrhoea treatment regimens: cost-effectiveness and impact on ceftriaxone use of five hypothetical strategies compared with standard care in England sexual health clinics" on behalf of the authors who remain responsible for the accuracy and appropriateness of the content. The same standards for ethics, copyright, attributions and permissions as for the article apply. Eurosurveillance is not responsible for the maintenance of any links or email addresses provided therein.

Strategy	Total cost [a]	Number of antibiotics used to treat NG			Number of optimal treatments[b]	Number of sub-optimal treatments[c]	Number of MEITR[d]	Number of treatment failures[e]
		Ceftriaxone	Azithromycin	Ciprofloxacin				
Standard care	£622,155<br>832,001.5 EUR	8,467	8,467	0	8,018	449	N/A	0
A) Dual therapy single POCT - Ciprofloxacin	£861,471<br>1,152,036 EUR	8,467	2,754	5,713	8,266	201	57	0
B) Dual therapy dual POCT - Azithromycin Ciprofloxacin	£891,674<br>1,192,427 EUR	8,467	7,947	351	8,455	12	58	0
C) Dual therapy dual POCT - Ciprofloxacin Azithromycin	£885,829<br>1,184,610 EUR	8,467	2,585	5,713	8,409	58	220	0
D) Monotherapy Azithromycin	£714,111<br>954,973.3 EUR	529	7,947	520	8,289	178	80	9

E) Monotherapy Ciprofloxacin	£754,263<br>1,008,668 EUR	2,809	2,754	5,713	8,266	201	57	55

SC, Standard Care; AMR, antimicrobial resistance; POCT, point-of-care test; NG, *Neisseria gonorrhoeae*; MEITR, missed earlier intended treatment regimen

[a] GBP costs were converted to Euros using a historic currency conversion of an average of 366 days from the 1st July 2015 to 30th June January 2016 [33]. For this time period, 1 GBP = 1.34 Euros, and 1 Euro = 0.75 GBP.

[b]'optimal' refers to a treatment regimen which cures the NG infection and does not contain any antibiotic against which there is resistance

[c]'sub-optimal' refers to a treatment regimen which contains antibiotics against which there is NG resistance - if the treatment is a monotherapy it will result in treatment failure

[d]'missed earlier intended treatment regimen' (MEITR) refers to a treatment regimen which cures the NG infection and does not contain any antibiotic against which there is resistance, but a treatment regimen was used when an earlier intended treatment regimen would have provided optimal treatment – a MEITR is due to a false-resistant AMR-POCT result

[e]'treatment failure' refers to failure to cure an NG infection due to resistance to an antibiotic given as monotherapy and is due to a false-susceptible AMR-POCT result

Supplementary Table S6. Total costs, treatments used, and treatment outcomes for SC and AMR-POCT strategies; MSW (n=8,467)

This supplementary material is hosted by Eurosurveillance as supporting information alongside the article “Antimicrobial resistance point-of-care testing for gonorrhoea treatment regimens: cost-effectiveness and impact on ceftriaxone use of five hypothetical strategies compared with standard care” on behalf of the authors who remain responsible for the accuracy and appropriateness of the content. The same standards for ethics, copyright, attributions and permissions as for the article apply. Eurosurveillance is not responsible for the maintenance of any links or email addresses provided therein.

Strategy	Total cost[a]	Number of antibiotics used to treat NG			Number of optimal treatments[b]	Number of sub-optimal treatments[c]	Number of MEITR[d]	Number of treatment failures[e]
		Ceftriaxone	Azithromycin	Ciprofloxacin				
Standard care	£623,698<br>834,064.9 EUR	8,488	8,488	0	8,259	229	N/A	0
A) Dual therapy single POCT - Ciprofloxacin	£862,495<br>1,153,406 EUR	8,488	1,740	6,748	8,407	81	68	0
B) Dual therapy dual POCT - Azithromycin Ciprofloxacin	£894,126<br>1,195,706 EUR	8,488	8,181	244	8,482	6	68	0
C) Dual therapy dual POCT - Ciprofloxacin Azithromycin	£887,037<br>1,186,225 EUR	8,488	1,677	6,748	8,453	35	127	0
D) Monotherapy Azithromycin	£711,726	312	8,181	307	8,422	66	83	5

	951,783.9 EUR							
E) Monotherapy Ciprofloxacin	£734,084<br>981,683 EUR	1,774	1,740	6,748	8,407	81	68	34

SC, Standard Care; AMR, antimicrobial resistance; POCT, point-of-care test; NG, *Neisseria gonorrhoeae*; MEITR, missed earlier intended treatment regimen; MSW, men-who-have-sex-with-women; N/A, Not Applicable

[a] GBP costs were converted to Euros using a historic currency conversion of an average of 366 days from the 1st July 2015 to 30th June January 2016 [33]. For this time period, 1 GBP = 1.34 Euros, and 1 Euro = 0.75 GBP.
[b]'optimal' refers to a treatment regimen which cures the NG infection and does not contain any antibiotic against which there is resistance
[c]'sub-optimal' refers to a treatment regimen which contains antibiotics against which there is NG resistance - if the treatment is a monotherapy it will result in treatment failure
[d]'missed earlier intended treatment regimen' (MEITR) refers to a treatment regimen which cures the NG infection and does not contain any antibiotic against which there is resistance, but a treatment regimen was used when an earlier intended treatment regimen would have provided optimal treatment – a MEITR is due to a false-resistant AMR-POCT result
[e]'treatment failure' refers to failure to cure an NG infection due to resistance to an antibiotic given as monotherapy and is due to a false-susceptible AMR-POCT result
